# MPP8 Facilitates DNA DSB Repair via NHEJ to Promote Chemoresistance of TNBC Independent of Methyl‐H3K9 Binding

**DOI:** 10.1002/advs.78157

**Published:** 2026-10-06

**Authors:** Rupa Kumari, Jia Fang

**Affiliations:** ^1^ Department of Pharmacology & Therapeutics Roswell Park Comprehensive Cancer Center Buffalo NY USA

**Keywords:** chemoresistance, DNA DSB repair, MPP8, NHEJ, TNBC

## Abstract

While cytotoxic chemotherapy remains the backbone of systemic treatments for triple‐negative breast cancer (TNBC), patients frequently develop resistance and recurrence, leading to poor clinical outcomes. However, molecular mechanisms underlying chemotherapy resistance are enigmatic. In this study, a pivotal epigenetic regulator MPP8 is characterized as a key player in TNBC chemoresistance. MPP8 depletion in TNBC cells significantly increases levels of cleaved‐ PARP1 and Caspase3 and reduces clonogenic survival upon doxorubicin or IR treatment, which are accompanied by a drastically delayed DNA damage repair progression and γH2AX clearance. Mechanistically, MPP8 robustly interacts with DNAPK complex and promotes DNA double‐strand break (DSB) repair via non‐homologous end joining (NHEJ) pathway independent of its epigenetic functions. Upon DNA damage, MPP8 is dynamically recruited to DNA DSBs, where it facilitates the recruitment of ligase complex and assembly of NHEJ apparatus to expedite DNA repair progression. Correspondingly, MPP8 knockdown resensitizes TNBC to doxorubicin treatment in murine orthotopic xenograft models. Together, these findings not only highlight a crucial non‐canonical function of MPP8 in DSB repair as a novel NHEJ accessary factor but also suggest a potential strategy to improve efficacy and outcomes of standard TNBC chemotherapy based on MPP8 expression levels.

## Graphical abstract

Chemoresistance is often linked to elevated DNA repair. MPP8 is found to promote breast cancer chemoresistance as a novel NHEJ accessory factor independent of its epigenetic functions. Through multiple protein interactions, MPP8 facilitates association of DNAPK and ligase complexes to promote DNA repair progression. MPP8 depletion resensitizes breast cancer cells to genotoxic agents, suggesting a novel therapeutic strategy.

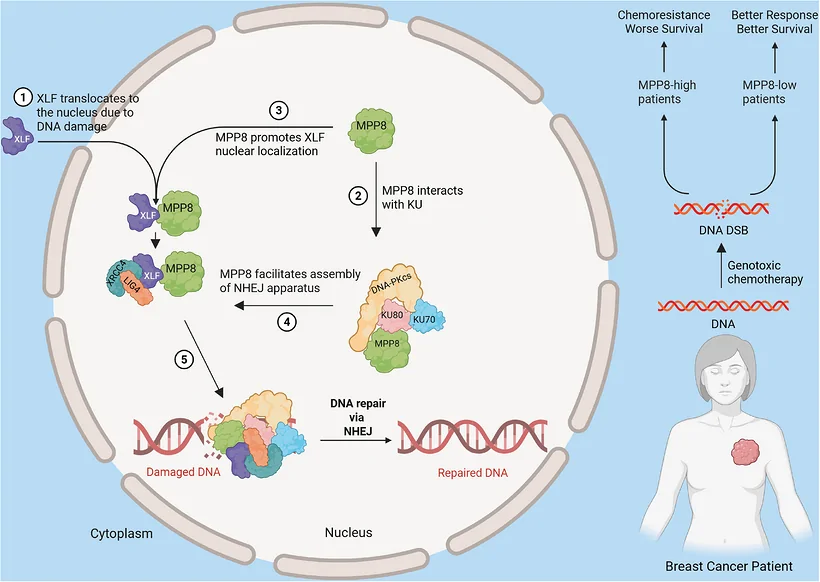

## 1 Introduction

Triple‐negative breast cancer (TNBC) is characterized by the absence of estrogen and progesterone receptors expression and human epidermal growth factor receptor‐2 amplification and is associated with high relapse rate and the worst outcomes of all breast cancers [1]. Despite recent progress in the development of targeted strategies and immunotherapies, genotoxic chemotherapy remains the backbone of systemic treatment for TNBC in both the neoadjuvant and the adjuvant settings. Although TNBC is intrinsically sensitive to chemotherapies and has a better response rate compared with other breast cancer subtypes, about 50% of TNBC patients evolve resistance and recurrence in 3 years, leading to a dismal median overall survival of 10.2 months with a 5‑year survival rate of 65% for regional tumors and 11% for metastatic diseases [2]. Additionally, current chemotherapy regimens have reported limited durability of response and an unfavorable toxicity profile. Therefore, further understanding of mechanisms underlying chemoresistance is important to improve current therapeutic options for TNBC.

Conventional genotoxic agents such as anthracyclines and platinum induce DNA damage and double‐strand breaks (DSBs) through different mechanisms. Multiple TNBC chemotherapy regimens include doxorubicin [1, 2], which induces DNA DSBs by DNA intercalation and Topo II poisoning [3]. As one of the most deleterious DNA lesions, DNA DSBs trigger different DNA damage responses (DDR) and are mainly repaired by two distinct canonical pathways—homologous recombination (HR) and non‐homologous end joining (NHEJ). While HR requires a sister chromatid template in S/G2 phase and has a major role to repair DSBs caused by stalled/collapsed replication forks, NHEJ rejoins the broken DSB ends in all cell cycle phases [4]. The high prevalence of HR deficiency in TNBC, including germline BRCA mutations and BRCAness exposes therapeutic vulnerabilities and has resulted in wealthy clinical successes [2]. In contrast, spontaneous mutations in NHEJ factors are very rare in human [4]. Intriguingly, profiling different clinic topoisomerase inhibitors with 48 isogenic DT40 mutant cells deficient in DNA repair found that NHEJ is specifically required for the repair of DSBs induced by Topo II inhibitors such as doxorubicin [5], suggesting that NHEJ could also play pivotal roles in chemotherapy response. However, the importance of NHEJ in chemoresistance of TNBC is understudied and remains elusive.

In eukaryotes, DSB repair operates in both euchromatin and heterochromatin regions, which are decorated by different permissive and repressive histone modifications, respectively [6]. As a heterochromatin mark, histone H3K9 trimethylation (H3K9me3) functions through specific methyl‐binding proteins and has been implicated in DDR and DSB repair [7]. We have previously characterized MPP8 as a H3K9me3 binding protein which coordinates with various chromatin‐modifying enzymes for tumor suppressor gene silencing and epithelial‐mesenchymal transition (EMT) in TNBC cells [8, 9]. MPP8 also serves as a central player in human silencing hub (HUSH) complex which represses integrated transgenes and retrotransposon elements (TEs) including LINE‐1s [10, 11, 12]. In AML, loss of MPP8 causes LINE‐1 reactivation and retrotransposition which trigger genome instability and cell cycle arrest via p53/p21 and abrogate cancer development [13]. In MPP8‐depleted somatic cells, LINE‐1 de‐repression also produces dsRNAs to induce interferon‐stimulated genes and inflammatory reactions and elevates DNA damage [14]. Although these studies suggest that MPP8 may be involved in DDR, the emerging role of MPP8 in DSB repair and its implications in TNBC are completely unknown.

Here, we show that MPP8 promotes chemoresistance of TNBC cells to doxorubicin by augmenting DNA DSB repair via NHEJ pathway independent of its H3K9me3 binding ability. Mechanistically, MPP8 interacts with DNAPK complex and facilitates stabilization of NHEJ repair apparatus at DSBs by promoting the recruitment of XLF and ligase complex after DNA damage. Loss of MPP8 significantly delays DNA DSB repair progression via NHEJ pathway and thus resensitizes TNBC cells to doxorubicin treatment in vitro and in xenograft models. Together, our findings not only characterize MPP8 as an unreported NHEJ factor but also suggest that MPP8 level may serve as a predictor for chemotherapy outcome of TNBC.

## 2 Results

### 2.1 MPP8 Promotes Resistance of TNBC Cells to Chemotherapy Drug

In previous studies, we have characterized MPP8 as a methyl‐H3K9 binding protein and demonstrated that MPP8 contributes to tumor suppressor gene silencing and EMT in breast and prostate cancer cells via H3K9me3 binding [8, 9, 15]. To evaluate the prognostic significance of MPP8 in breast cancer, we analyzed the RNA‐Seq dataset from a recent pan‐cancer study [16]. Consistent with pro‐oncogenic function of MPP8 we reported, patients with elevated MPP8 expression trend towards worse survival (*n* = 1089) although this association is not statistically significant (*p* = 0.38, Figure S1A). We further examined another transcriptomic database containing 55 independent DNA microarray datasets (*n* = 2032) which also include the treatment information [17]. Among four probes, we focused on 225041_at because it displayed a similar trend on survival (*p* = 0.098, Figure 1A). Intriguingly, the correlation between elevated MPP8 expression and worse survival became statistically significant when we stratified patients who received chemotherapy (*p* = 0.03), whereas such a significance was not observed in patients who did not (*p* = 0.2, Figure 1B,C), suggesting that MPP8 could be involved in chemotherapy response of breast cancer patients.

**FIGURE 1:**
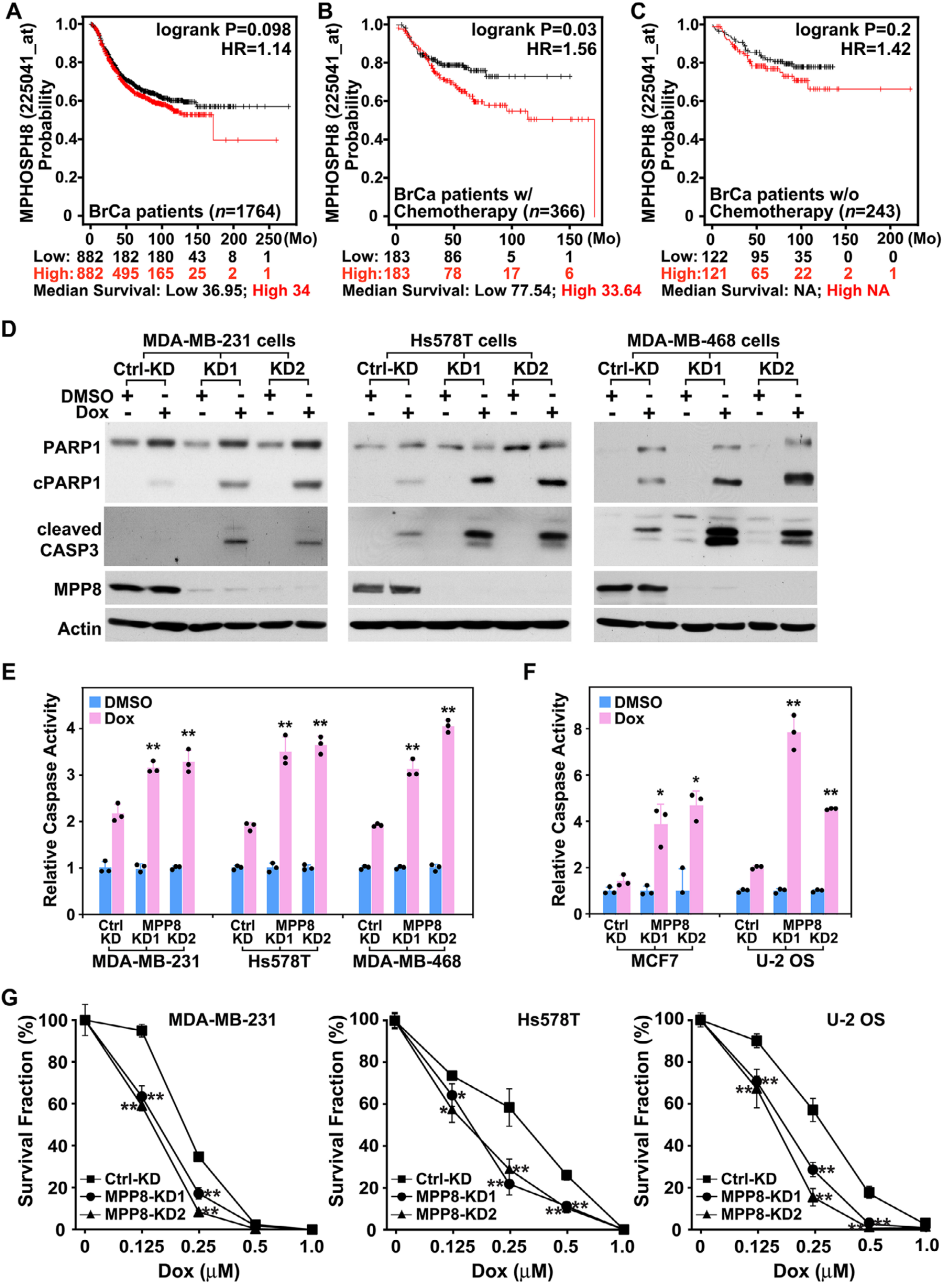
MPP8 promotes chemoresistance of TNBC cells to doxorubicin. (A–C) Kaplan–Meier plots for overall survival of breast cancer patients stratified by MPP8 expression level with *p*‐values from univariate (log‐rank). (A) All patients. (B) Patients received chemotherapy. (C) Patients without chemotherapy. (D) Western blot analysis of whole cell extracts (WCE) derived from different control‐ and MPP8‐ KD TNBC cells after DMSO or Dox treatment using indicated antibodies. (E,F) Caspase3 activities (DEVDase) were measured in vitro using cell lysates derived from indicated control‐ and MPP8‐ KD TNBC cells (E) and other tumor cells (F) after DMSO or Dox treatment. Results are mean±s.d. from three independent samples while DMSO‐treated cells were normalized as 1. *p < 0.05, **p < 0.01. (G) Clonogenicity assays of indicated control‐ and MPP8‐ KD tumor cells with increasing doses of Dox. Colonies were quantified after 14 days while colony numbers without Dox were normalized as 100%. Results are mean±s.d. from three independent experiments. *p < 0.05, **p < 0.01.

Chemotherapy is the frontline system treatment for most patients with stage II‐III TNBC and many regimens include doxorubicin (Dox) due to its efficacy [1]. To assess the role of MPP8 in chemotherapy response, we stably knocked down (KD) MPP8 in three TNBC cell lines with different genetic backgrounds, including MDA‐MB‐231 (D3H2LN‐Luc subline), Hs578T and MDA‐MB‐468 and examined their response to Dox treatment. Comparing with control‐KD, all MPP8‐KD cells showed a drastic increase of cleaved‐ PARP1 and Caspase3 levels on western blots (Figure 1D). A significantly increased Caspase3 activity was also readily detected (Figure 1E), suggesting that MPP8 could mitigate Dox‐induced apoptosis. This possibility was confirmed by flow cytometry analysis of Annexin V‐stained cells, in which all MPP8‐KD cells displayed a significantly increased apoptotic population upon Dox treatment (Figure S1B). To test whether it is cell‐type specific, we generated control‐ and MPP8‐ KD MCF7 and U‐2 OS cells for the same set of assays and obtained similar results (Figure 1F and Figure S1C–E), indicating that MPP8‐mediated anti‐apoptotic effect is a general phenomenon. Furthermore, we carried out clonogenic survival assays to determine the survival and proliferative potential of cells with or without MPP8 after Dox treatment. Similarly, all MPP8‐KD cells showed a significantly reduced reproductive integrity to form large colonies comparing with their control‐KD counterparts (Figure 1G). Together, these results demonstrate that MPP8 alleviates apoptosis induced by doxorubicin in different cancer cells and could potentially promote resistance to such a treatment.

### 2.2 MPP8 Facilitates Repair of DNA Double‐Strand Breaks (DSBs)

As one major mechanism of action by doxorubicin is intercalating double‐strand DNA to stall replication forks and induce DSBs [18], we reasoned that MPP8 could be involved in DNA damage response (DDR). To test this possibility, we utilized the alkaline comet assay and measured DNA damage in control‐ and MPP8‐ KD MDA‐MD‐231 cells after Dox treatment. As indicated in Figure 2A,B, control‐KD and MPP8‐KD cells showed same amount of damaged DNA before and right after Dox treatment, indicating that MPP8 loss does not affect basal or acute DDR. However, a significant delay in late repair progression was readily detected 24 h after Dox treatment. As the alkaline comet assay detects both single and double strain breaks, we further employed the neutral comet assay and more specifically measured DSBs in the same set of cells after γ‐irradiation (IR). Similarly, basal DSB level and the pattern of acute repair response are comparable in control‐KD and MPP8‐KD cells, whereas the clearance of DSBs 24 h after IR is significantly slower in MPP8‐KD cells (Figure 2C,D). To validate these results, we also examined γH2AX level in different control‐ and MPP8‐ KD cells upon Dox or IR treatment. After IR, immunostaining detected a comparable amount of γH2AX accumulation in control‐KD and MPP8‐KD MDA‐MB‐231 cells. However, resolution of formed γH2AX foci are significantly slower in MPP8‐KD cells 24 h after IR (Figure 2E,F). Similarly delayed clearance of formed γH2AX foci were also detected in two additional MPP8‐KD TNBC cell lines MDA‐MB‐468 and Hs578T (Figure S2A–D). Such phenotypes are clearly not cell type specific as U‐2 OS cells also displayed similar γH2AX formation and resolving patterns (Figure 2G and Figure S2E). On western blots, control‐KD and MPP8‐KD MDA‐MB‐231 and U‐2 OS cells showed an increased accumulation of γH2AX after Dox treatment. Similarly, γH2AX clearance was significantly impeded in MPP8‐KD cells at late stage (Figure 2H). Moreover, MPP8‐KD MDA‐MB‐231, Hs578T and U‐2 OS cells displayed significantly reduced clonogenic survival in response to increased IR doses (Figure 2I,J and Figure S2F), indicating an increased accumulation of unrepaired DSBs in these cells. Together, these findings support an unreported role of MPP8 in DNA DSB repair progression.

**FIGURE 2:**
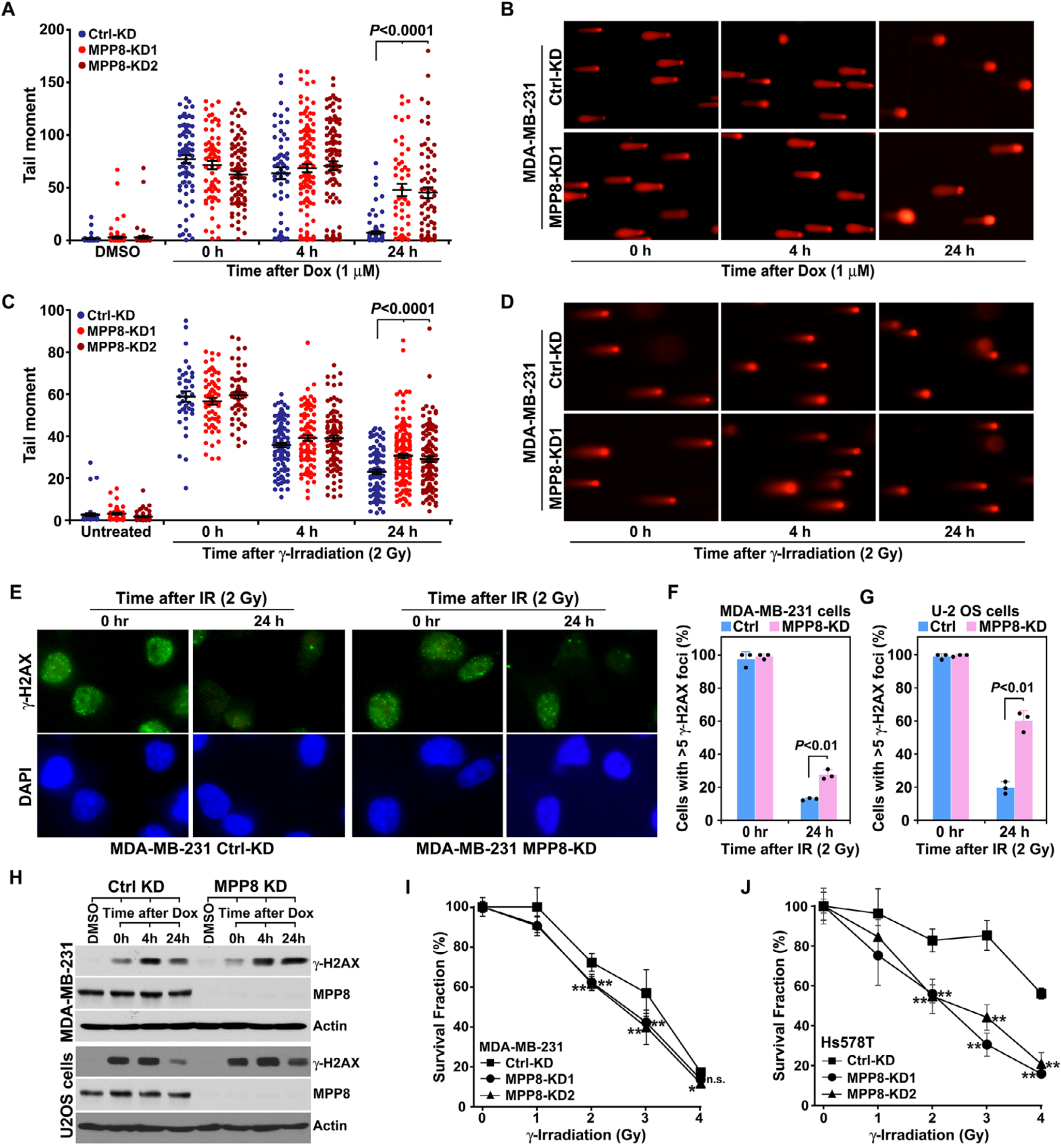
MPP8 promotes repair of DNA DSBs. (A) Dox‐induced DNA damage in control‐ and two MPP8‐ KD MDA‐MB‐231 cells were quantified by the tail moment in the alkaline comet assay at indicated time points. Error bars are ±SEM of assayed cells (*n* > 200). (B) Representative images of the tail moment in control‐ and MPP8‐ KD cells at indicated time points. (C) IR‐induced DNA damage in control‐ and MPP8‐ KD MDA‐MB‐231 cells were quantified in the neutral comet assay (mean±SEM, *n* > 200). (D) Representative images of the tail moment in control‐ and MPP8‐ KD cells in the neutral comet assay. (E) Immunostaining of γH2AX (green) and DAPI (blue) in control‐ and MPP8‐ KD MDA‐MB‐231 cells 0 and 24 h after IR treatment. (F,G) Quantification of control‐ and MPP8‐ KD MDA‐MB‐231 (F) and U‐2 OS (G) cells with γH2AX foci (>5/cell) after IR treatment. Results are mean±s.d. from three independent experiments and 150–200 cells were analyzed for each group. (H) Western blot analysis of WCE derived from control‐ and MPP8‐ KD cells after DMSO or Dox treatment at different time points using indicated antibodies. (I,J) Clonogenicity assays of control‐ and MPP8‐ KD MDA‐MB‐231 (I) and Hs578T (J) cells with increasing doses of IR. Colonies were quantified after 14 days and colony numbers without IR were normalized as 100%. Results are mean±s.d. from three independent experiments. *p < 0.05, **p < 0.01, n.s. not significant.

### 2.3 MPP8 Promotes DNA DSB Repair Through NHEJ Pathway

In mammals, DNA DSBs are mainly repaired via HR and NHEJ pathways with different sets of DDR sensors, mediators, and effectors [4]. To determine how is MPP8 involved in DNA DSB repair, we purified MPP8 protein complex from the nuclear extract fraction derived from suspension‐cultured HeLa‐S3 cells stably expressing Flag‐HA‐MPP8 using a tandem‐tag affinity approach [19]. Mass‐Spec analysis of excised protein bands from SDS‐PAGE gel identified prior reported MPP8‐interacting proteins such as TASOR [12], PPHLN1 [12] and NP220 [20]. Intriguingly, it also identified DNAPK complex composed of KU70, KU80 and DNA‐PKcs (Figure 3A). IP‐western analysis further confirmed that exogenous Flag‐ KU70 or KU80 interacts with endogenous and exogenous MPP8, and these interactions remain stable after extensive washings with 1.0 M NaCl (Figure S3A–C). To determine whether these interactions are affected by DDR, we treated KU70 or KU80‐transfected cells with Dox and carried out same IP‐western analysis at different time points. As shown in Figure S3D, MPP8‐KU70/80 interactions increased moderately 2 h after Dox treatment and gradually reduced to basal level after 24 h. suggesting that Dox‐induced DDR could further enhance these interactions. Given that KU70 and KU80 bind to DNA and RNA robustly, we also examined if DNA and RNA could be involved in MPP8‐KU70/80 interactions. Intriguingly, pre‐treatment of cell lysates with DNase I, RNase A or both before IP showed no obvious effect (Figure S3E,F), indicating that these interactions are mostly mediated at the protein level. Considering that KU70‐KU80 heterodimer is resistant to separation unless subjected to high salt plus strong ionic detergents [21, 22], we further challenged MPP8‐KU70/80 interactions under these conditions. As indicated in Figure 3B, Flag‐KU80 interacts with endogenous MPP8 much stronger than Flag‐KU70 after extensive washings with 1.5 M NaCl, even though Flag‐KU70 showed better IP efficiency (lanes 1&4). Additional washes with detergent‐rich mixed micelle buffer (MMB) [22] significantly reduced KU70‐MPP8 binding while only slightly impacted KU80‐MPP8 interaction (lanes 2&5). As previously reported [22], extensive washing with high salt (1.0 M KCl) and strong ionic detergent sodium deoxycholate (SDC) simultaneously dissociated KU70‐KU80 dimer, however, KU80‐MPP8 interaction largely sustained (lanes 3&6). Therefore, these results suggest that MPP8 interacts with KU70/KU80 heterodimer and DNAPK complex via its robust binding to KU80 and such interactions could be further enhanced upon DNA damage.

**FIGURE 3:**
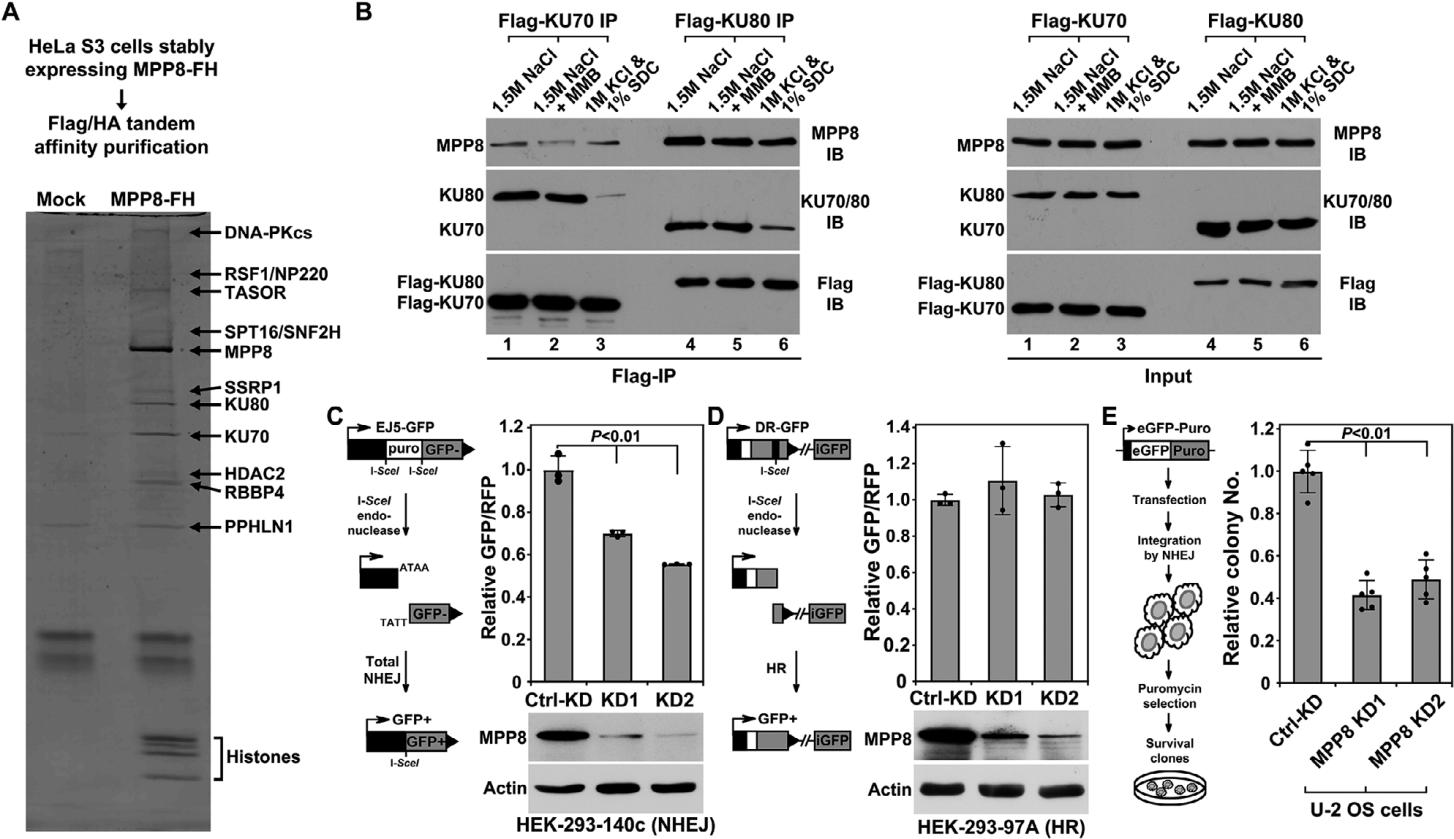
MPP8 promotes DNA DSB repair via NHEJ pathway. (A) Coomassie blue staining of an SDS‐PAGE gel resolving affinity‐purified MPP8 complex. Indicated protein bands were excised and identified by Mass‐Spec. (B) IP‐western blot analysis of exogenous expressed Flag‐ KU70/KU80 interactions with endogenous MPP8 or KU80/KU70 under different indicated conditions (left). Inputs were also analyzed (right). (C) Schematic of NHEJ repair using EJ5‐GFP reporter (left) and quantification of NHEJ activity in control‐ and two stable MPP8‐ KD HEK‐293‐140c reporter cells (right). I‐SceI transfection efficiency was normalized by co‐transfected mCherry while NHEJ activity in control‐KD cells was normalized as 1. Results are mean±s.d. from three independent experiments. (D) Schematic of HR repair using DR‐GFP reporter (left) and quantification of HR activity in control‐ and two stable MPP8‐ KD HEK‐293‐97A reporter cells (right). Results are mean±s.d. from three independent experiments while HR activity in control‐KD cells was normalized as 1. (E) Schematic of random plasmid integration assay (left) and quantified results in control‐ and two stable MPP8‐ KD U‐2 OS cells (right). Results are mean±s.d. from three independent experiments, while colony number of control‐KD cells were normalized as 1.

The strong interaction between MPP8 and DNAPK complex, the core component of NHEJ pathway prompted us to test if MPP8 is involved in DNA DSB repair via NHEJ. First, we stably knocked down MPP8 in a well‐established 293‐140c cell line containing an EJ5‐GFP reporter in which successful repair of I‐SceI endonuclease‐induced DSBs via NHEJ pathway can be monitored by restored GFP expression [23]. After I‐SceI expression, a significantly reduced NHEJ repair activity was readily detected by flow cytometry analysis of MPP8‐KD 293‐140c reporter cells (Figure 3C). Next, we generated MPP8‐KD 293–97A cells harboring a DR‐GFP reporter in which repair of I‐SceI‐induced DSB via HR pathway can be similarly examined by GFP expression [23]. In sharp contrast, control‐ and MPP8‐ KD 293‐97A reporter cells showed similar HR repair activities (Figure 3D), suggesting that MPP8‐mediated DSB repair is limited to NHEJ pathway. Since EJ5‐GFP reporter measures total NHEJ activities including cNHEJ (canonical) and altNHEJ (alternative) [24], we further transfected control‐ and MPP8‐ KD U‐2 OS cells with a linearized plasmid expressing an eGFP‐Puro fusion protein and measured its random integration into the genome mediated by cNHEJ. A significant reduction of random plasmid integration was readily detected in both PP8‐KD cells (Figure 3E). Therefore, these findings together demonstrate that MPP8 contributes to DNA DSB repair via cNHEJ pathway.

### 2.4 MPP8 Promotes NHEJ DSB Repair Independent of Methyl‐H3K9 Binding

Prior report revealed that multiple DSB repair proteins interact with KU70/80 through a conserved KU binding motif (KBM) [25]. However, we could not identify any KBM in MPP8. To map the KU‐interacting domain in MPP8, we co‐expressed different Flag‐MPP8 deletions with HA‐KU80 for IP‐western analysis. As shown in Figure 4A, MPP8 C‐terminal domain (525‐860aa) is not only necessary but also sufficient to interact with KU80, whereas the N‐terminal chromo‐domain and middle parts of MPP8 failed to do so. Moreover, Dox treatment of transfected cells before IP showed similar interaction pattern (Figure S4A), confirming that the chromodomain‐containing MPP8 N‐terminus is indeed dispensable for MPP8‐KU80 interaction with or without DDR. Given that MPP8 most likely interacts with KU80 at protein level and the crystal structure of MPP8 C‐terminus (565‐860aa) has been reported recently [26], we utilized Alphafold3 [27] and generated a predicted atomic model of MPP8_525‐860_‐KU80 protein complex solely with their amino acid sequences (Figure S4B). Consistent with our domain mapping data, protein contact analysis [28] (distance 8) predicted comprehensive interactions involving multiple regions of MPP8 and KU80 (Figure S4C), although the stringent cutoff (distance 3) suggests that more confident interactions are likely mediated between MPP8_741‐826_ and KU80_299‐582_ (Figure S4D). To further determine the functional role of KU80 binding domain in MPP8‐mediated DSB repair, we stably rescue expressed MPP8‐wt, MPP8‐W80A, MPP8_1‐525_ or MPP8_525‐860_ in MPP8‐KD 293‐140c NHEJ reporter cells. Consistent with their KU80 binding abilities, MPP8_525‐860_ significantly restored NHEJ repair capacity in MPP8‐KD cells while MPP8_1‐525_ failed to do so. Meanwhile, MPP8‐W80A, a chromodomain mutant deficient in H3K9me3 binding, showed same NHEJ repair capacity as MPP8‐wt (Figure 4B). In contrast, MPP8‐KD and rescue expression of different MPP8 have no impact on DSB repair via HR pathway in 293‐97A reporter cells (Figure 4C). These results not only support that MPP8 interacts with KU80 to promote NHEJ DSB repair but also confirm that NHEJ repair mediated by MPP8 is independent of its H3K9me3 binding ability and EMT activity, even though EMT has been previously associated with DNA DSB repair [29].

**FIGURE 4:**
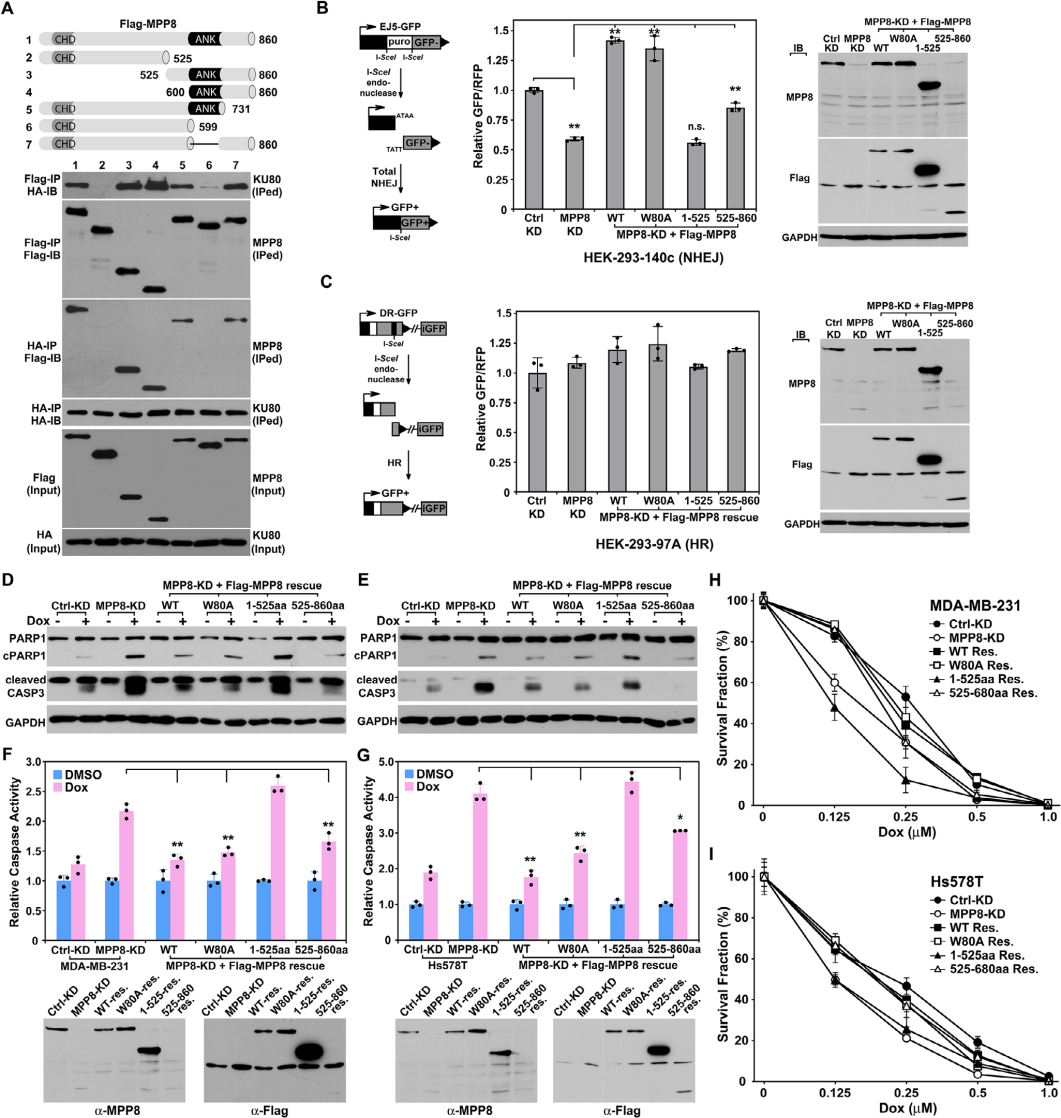
MPP8 promotes NHEJ repair and chemoresistance via its C‐terminal domain. (A) Different Flag‐MPP8 deletions (top) were co‐expressed with HA‐KU80 in MPP8‐KD 293T cells followed by Flag or HA IP‐western analysis using indicated antibodies. Inputs were also analyzed. (B) Schematic of NHEJ repair using EJ5‐GFP reporter (left) and quantification of NHEJ activity in control‐KD, MPP8‐KD, and different stable rescue HEK‐293‐140c reporter cells (middle). Results are mean±s.d. of three independent experiments and NHEJ activity in control‐KD cells was normalized as 1. **p < 0.01. MPP8 expression was confirmed by western blot (right). (C) Schematic of HR repair using DR‐GFP reporter (left) and quantification of HR activity in control‐KD, MPP8‐KD, and different rescue HEK‐293‐97A reporter cells (middle). Results were normalized and analyzed similarly while MPP8 expression was confirmed by western blot (right). (D,E) Western blot analysis of control‐KD, MPP8‐KD, and different rescue MDA‐MB‐231 cells (D) and Hs578T cells (E) after DMSO or Dox treatment with indicated antibodies. (F,G) Caspase3 activity of cell lysates derived from same set of MDA‐MB‐231 cells (F) and Hs578T cells (G) after DMSO or Dox treatment. Results are mean±s.d. from three independent experiments and DMSO group was normalized as 1. **p < 0.01. MPP8 expression in different cells was confirmed by western blot (bottom). (H,I) Clonogenicity assays of same set of MDA‐MB‐231 cells (H) and Hs578T cells (I) with increasing doses of Dox. Colonies were stained after 14 days while colony number from DMSO group were normalized as 100%. Results are mean±s.d. from three independent experiments.

To further assess the importance of NHEJ repair by MPP8 in chemoresistance of TNBC cells, we stably rescue expressed same set of Flag‐MPP8s in MPP8‐KD MDA‐MB‐231 and Hs578T cells. As shown in western blots, elevated levels of cleaved PARP1 and Caspase3 reduced to normal by rescue expressed MPP8‐wt, MPP8‐W80A or MPP8_525‐860_, but not MPP8_1‐525_ (Figure 4D,E). MPP8‐wt, MPP8‐W80A or MPP8_525‐860_ also significantly mitigated elevated Caspase3 activity in MPP8‐KD cells while MPP8_1‐525_ showed no effect (Figure 4F,G). We also assessed clonogenicity of different MPP8‐ KD and rescue cells by exposing them to increased doses of Dox. Similarly, rescue expression of MPP8‐wt, MPP8‐W80A or MPP8_525‐860_ largely restored Dox resistance of MPP8‐KD MDA‐MB‐231 and Hs578T cells, whereas expression of MPP8_1‐525_ failed to do so. (Figure 4H,I). Together, these results demonstrate that MPP8 promotes chemoresistance of TNBC cells mostly via its C‐terminal domain (525‐860aa). Considering that this domain interacts with KU80 and is responsible for MPP8‐mediated NHEJ repair, we further conclude that MPP8 augments NHEJ DSB repair capacity to promote chemoresistance of TNBC cells via its C‐terminal domain independent of its canonical H3K9me3 binding ability.

### 2.5 MPP8 Facilitates Nuclear Translocation of XLF and Assembly of NHEJ Repair Complex

NHEJ is initiated at KU70/80 binding onto DSBs followed by DNA‐PKcs recruitment. Activated DNAPK complex then recruits ligase complex composed of LIG4, XRCC4, and XLF to seal DSBs [4]. The role of MPP8 in repair progression suggested a possibility that MPP8 could augment the function of ligase complex. While XRCC4 and LIG4 are nuclear proteins, XLF has been recently shown to localize in both the cytoplasm and the nucleus and translocate into the nucleus upon DNA damage [30, 31]. We thus tested if MPP8 affects XLF nuclear translocation. As shown in Figure 5A,B, immunostaining and quantification analysis confirmed that endogenous XLF localized in both the cytoplasm and the nucleus in Ctrl‐KD MDA‐MB‐231 cells and became enriched in the nucleus after Dox treatment. However, Dox‐induced XLF nuclear translocation is greatly reduced in MPP8‐KD cells. To validate these findings, we fractionated different cells for western blot analysis. Similarly, XLF was present in both cytoplasmic and nuclear fractions with a clear enrichment in the cytoplasm in both Ctrl‐KD and MPP8‐KD cells. 4 h after Dox treatment, XLF was enriched in the nuclear fraction derived from Ctrl‐KD cells but not MPP8‐KD cells (Figure 5C,D). In addition, we detected a similar reduction of DNA damage induced XLF nuclear translocation in MPP8‐KD U‐2 OS cells (Figure S5A,B). To delineate possible mechanism, we assessed MPP8‐XLF interaction by IP‐western analysis. Although Flag‐XLF or Flag‐XRCC4 marginally binds to endogenous MPP8 in DMSO‐treated cells, a moderate but clear XLF‐MPP8 interaction was readily detected upon Dox treatment, and this interaction significantly increased 4 h after treatment. In parallel, XRCC4‐MPP8 interaction remained at minimal level (Figure 5E). Furthermore, pre‐treatment of cell lysates with DNase I, RNase A or both before IP did not affect interaction patterns (Figure 5F), indicating that Dox‐induced MPP8‐XLF interactions mostly occur at the protein level. Therefore, we also modeled the structure of MPP8_525‐860_‐XLF protein complex using Alphafold3 with their primary sequences. As shown in Figure S5C,D, multiple predicted interactions localize between C‐terminuses of MPP8_525‐860_ and XLF, while high‐confident interactions are likely mediated by a group of very C‐terminal residues in MPP8 (mostly no overlap with KU80 interacting region) and several amino acids (169‐173aa) in the coil domain of XLF (Figure S5E). Given that MPP8 exclusively localizes in the nucleus [8, 32] while XLF dynamically translocated into the nucleus upon DNA damage, these results suggest that DNA damage could induce MPP8‐XLF protein interactions to facilitate XLF nuclear retention and expedite NHEJ repair progression.

**FIGURE 5:**
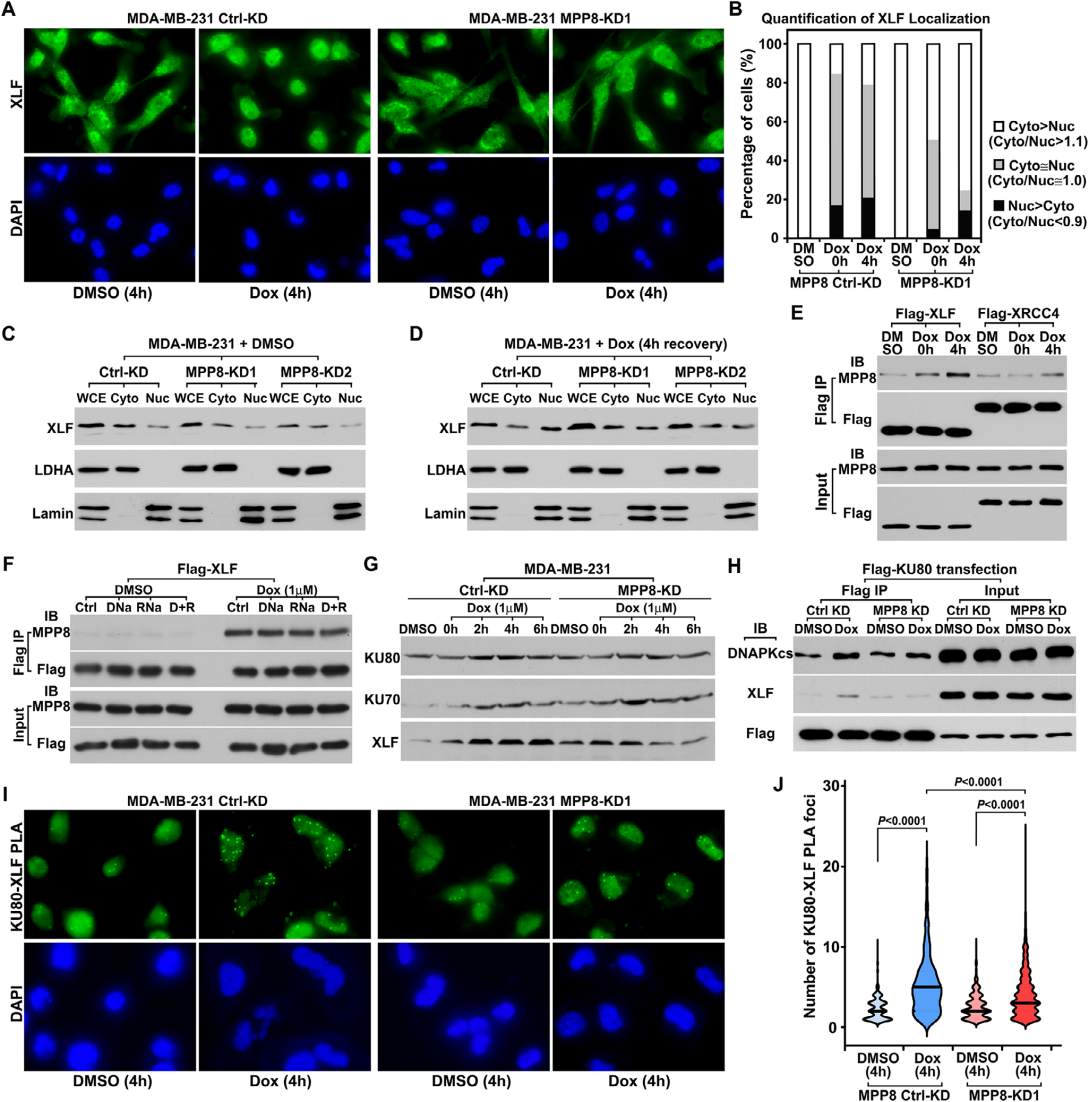
MPP8 facilitates XLF nuclear localization and assembly of NHEJ repair complex. (A) Immunostaining of XLF (green) and DAPI (blue) in control‐ (left) and MPP8‐ KD (right) MDA‐MB‐231 cells 4 h after DMSO or Dox treatment. (B) Quantification of XLF subcellular localization in control‐ and MPP8‐ KD MDA‐MB‐231 cells 4 h after DMSO or Dox treatment. The staining density of cytoplasmic and nuclear XLF in each cell was quantified while the ratios of cytoplasmic/nuclear XLF were indicated in the bar graph. 200–300 cells in each group were analyzed. (C,D) Western blot analysis of WCE, cytoplasmic, and nuclear fractions derived from control‐ and two stable MPP8‐ KD MDA‐MB‐231 cells 4 h after DMSO (C) or Dox treatment (D). LDHA and Lamin B serve as the cytoplasmic and nuclear markers, respectively. (E) IP‐western analysis of interactions of Flag‐tagged XLF or XRCC4 with endogenous MPP8 after DMSO or Dox treatment. (F) IP‐western analysis of interactions of Flag‐XLF with endogenous MPP8 after DMSO or Dox treatment. Cell lysates were pre‐treated with 100U/mL DNase I (DNa), 50 µg/mL RNase A (RNa), or both (D+R) before IP. (G) Western blot analysis of KU80, KU70, and XLF in the chromatin‐associated fractions derived from control‐KD and MPP8‐KD MDA‐MB‐231 cells at different time points after Dox treatment. (H) IP‐western analysis of interactions of Flag‐KU80 with endogenous DNA‐PKcs and XLF in control‐KD and MPP8‐KD 293T cells after DMSO or Dox treatment. Protein levels in input were also examined by western blot. (I) In situ KU80‐XLF interactions in control and MPP8‐KD MDA‐MB‐231 cells were measured by PLA before and 4 h after DMSO or Dox treatment (green). The nucleus was co‐stained by DAPI (blue). (J) Quantification of KU80‐XLF PLA foci. Results are mean±SEM while more than 300 cells were analyzed in each group.

The uncovered MPP8 interactions with both KU70/80 and XLF suggest that MPP8 may scaffold DNAPK and ligase complexes at DSBs to facilitate NHEJ repair. To test this possibility, we first isolated chromatin‐bound fractions from control‐KD and MPP8‐KD MDA‐MB‐231 cells after Dox treatment and examined chromatin association of KU70/80 and XLF at different time points by western blot. As expected, Dox treatment increased chromatin association of KU70/80 and XLF. Intriguingly, XLF chromatin association was greatly reduced in MPP8‐KD cells whereas KU70/80 chromatin association remained unchanged (Figure 5G), suggesting that MPP8 could promote XLF chromatin recruitment after DNA damage. Since XLF is recruited to DSB foci by DNAPK complex upon DNA damage, we next expressed Flag‐KU80 in control‐ and MPP8‐ KD cells and analyzed its interactions with XLF by IP‐western. As shown in Figure 5H, Dox treatment greatly strengthened KU80 interactions with both DNA‐PKcs and XLF. However, such enhanced KU80‐XLF interaction was largely impaired in MPP8‐KD cells while KU80‐DNA‐PKcs interactions were only moderately reduced. Furthermore, we assessed the association of KU80 and XLF using the proximal ligation assay (PLA) which allows detection of protein‐protein interactions in situ within the cell [33]. In control‐KD MDA‐MB‐231 cells, basal KU80‐XLF PLA foci number was low but increased drastically 4 h after Dox treatment, indicating a DNA damage‐induced assembly of NHEJ repair apparatus. In contrast, Dox‐induced increase of KU80‐XLF foci formation was greatly attenuated in MPP8‐KD cells (Figure 5I). PLA foci quantification confirmed that MPP8 depletion significantly reduced KU80‐XLF association induced by Dox treatment while it showed no impact on DMSO‐treated cells (Figure 5J). To validate the results, we also examined KU80‐XLF PLA foci formation in U‐2 OS cells. As expected, a drastic increase of KU80‐XLF association was readily detected in Ctrl‐KD cells after Dox treatment while such increase was largely diminished in MPP8‐KD cells (Figure S5F,G). Together, these results support a scenario in which MPP8 facilitates assembly of NHEJ repair apparatus to promote DSB repair, likely through its protein‐protein interactions with KU80 and XLF.

### 2.6 MPP8 is Recruited to DNA DSB Foci

Although our results suggest that MPP8 localizes at DSBs after DNA damage to facilitate association of DNAPK and ligase complexes, we could not detect MPP8 enrichment on γH2AX foci induced by DNA damage by immunostaining (data not shown), like many NHEJ factors. Therefore, we switched to a well‐established inducible homing endonuclease I‐PpoI system [34] and generated a U‐2 OS cell line stably expressing I‐PpoI fused with a destabilization domain (DD) and a mutant estrogen receptor nuclear localization domain (ER). In these cells, a transient and temporal induction of DNA DSBs at multiple defined genomic loci can be precisely controlled by sequential addition of Shield‐1 and 4‐hydroxytamoxifen (4‐OHT) (Figure 6A). To assess the role of MPP8 in repairing I‐PpoI‐induced DSBs, we also stably expressed shCtrl or shMPP8 in this established cell line. Using this system, we first examined DSB induction and repair dynamics, which were determined by qPCR over the I‐PpoI sites‐ using flanking primers. In control‐KD cells, cleavage of the I‐PpoI site within DAB1 gene increased after 4‐OHT addition and peaked at 3 h. At 6–12 h, most DSBs were repaired successfully, indicated by restoration of DNA level over the same I‐PpoI site. In MPP8‐KD cells. However, a significantly elevated accumulation of DSBs was readily detected 2 h after 4‐OHT addition, which kept increasing till 6 h before being gradually overwhelmed by repair progression (Figure 6B). Similarly, the increased DSB accumulation and a much‐delayed repair dynamics were also detected at two additional I‐PpoI sites within RYR2 and SLCO5A1 genes in MPP8‐KD cells (Figure 6C,D). Given that I‐PpoI‐induced DNA cutting and spontaneous DSB repair occur simultaneously while MPP8 depletion does not affect I‐PpoI expression and nuclear translocation (data not shown), we speculate that the increased DSB accumulation in MPP8‐KD cells is the result of a significantly delayed DSB repair process. Thus, these results further support that MPP8 augments DSB repair progression.

**FIGURE 6:**
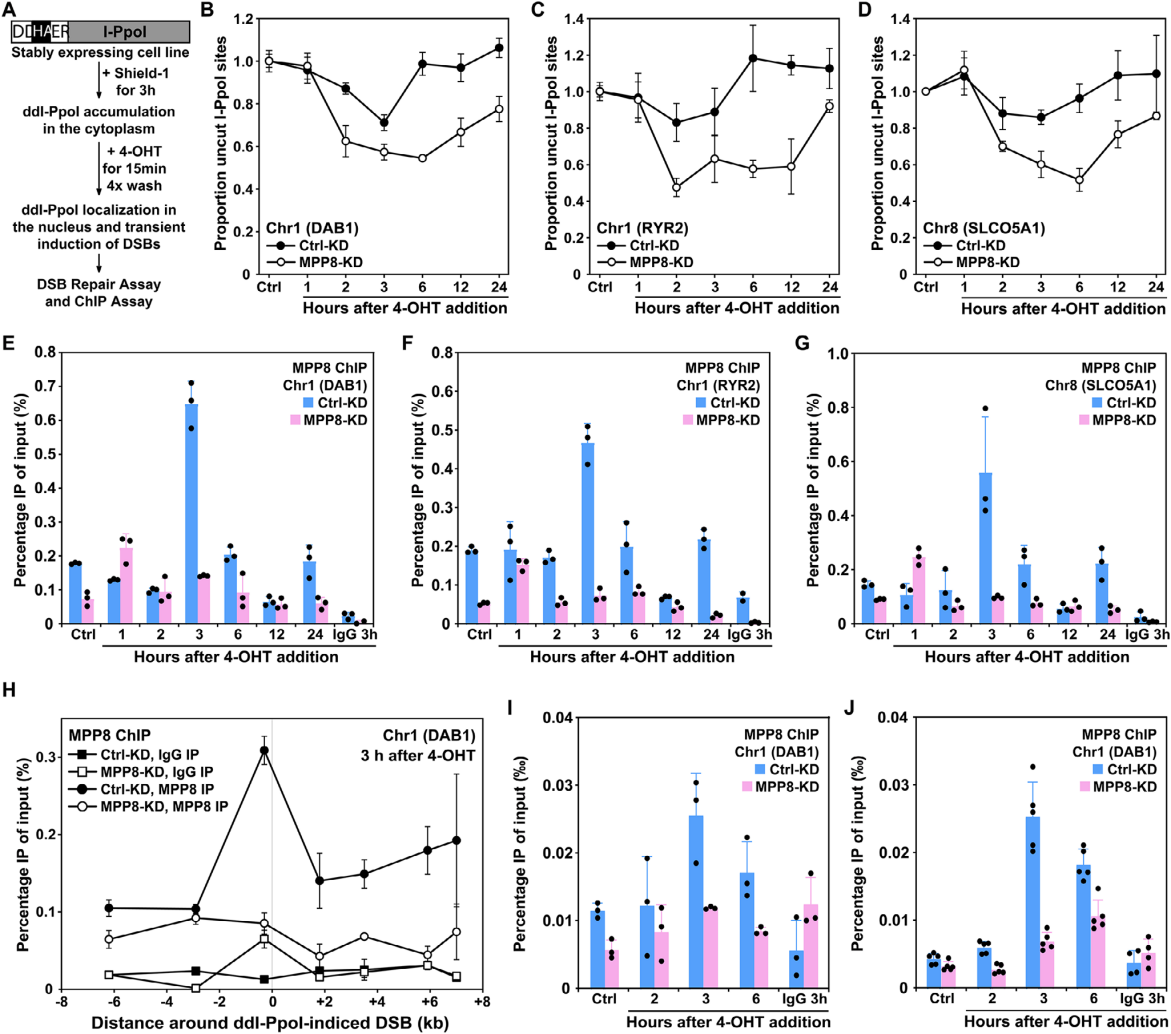
MPP8 is dynamically recruited to DNA DSB foci and facilitates the recruitment of ligase complex. (A) Experimental procedure to induce DSBs in ddI‐PpoI expressing U‐2 OS cells. (B–D) DSB induction and repair kinetics at the I‐PpoI sites in DAB1 (B), RYR2 (C), and SLCO5A1 (D) genes in control‐ and MPP8‐ KD cells. Results are mean±s.d. from three independent experiments while control treatment group was normalized as 1. (E–G) Quantified proportion of input DNA and MPP8 enrichment from ChIP‐qPCR analysis at the I‐PpoI sites in DAB1 (E), RYR2 (F) and SLCO5A1 (G) genes in control‐ and MPP8‐ KD cells. Results are mean±s.d. from three independent experiments. (H) ChIP‐qPCR analysis of MPP8 occupancy around the I‐PpoI site within DAB1 gene in control‐ and MPP8‐ KD cells 3 h after 4‐OHT addition. Results represent mean±s.d. from three independent experiments. (I,J) ChIP‐qPCR quantification of LIG4 occupancy at the I‐PpoI site within DAB1 (I) and RYR2 (J) genes in control‐ and MPP8‐ KD cells. Results are mean±s.d. from three independent experiments.

As the I‐PpoI system allows examination of protein localization around DSBs by chromatin IP (ChIP) assays, we next analyzed MPP8 enrichment at the I‐PpoI sites before and after DSB induction. In Ctrl‐KD cells, MPP8 localization at the I‐PpoI site in DAB1 gene was low before and right after 4‐OHT addition but increased sharply at 3 h and reduced to basal level after 6 h. In MPP8‐KD cells, such MPP8 enrichment is completely abolished (Figure 6E). A similar MPP8 recruitment pattern was also detected at the I‐PpoI sites in RYR2 and SLCO5A1 genes (Figure 6F,G). Therefore, these results clearly demonstrate that MPP8 is dynamically recruited to DSB foci induced by I‐PpoI during the repairing process. We further examined the distribution of MPP8 around the I‐PpoI site in DAB1 gene after 4‐OHT addition. Intriguingly, MPP8 was mostly enriched at the DSB site at 3 h time point and did not spread into the adjacent regions (Figure 6H and Figure S6A). Since our PLA results suggest that MPP8 facilitates assembly of NHEJ repair complex (Figure 5I,J), we also tested if MPP8 contributes to the recruitment of ligase complex using this system. As our XLF antibody did not work for ChIP (data not shown), we examined the recruitment of LIG4 in the same settings. Similar to MPP8, LIG4 was enriched at the I‐PpoI sites in DAB1 and RYR2 genes 3–6 h after 4‐OHT addition and gradually reduced to basal level in control‐KD cells. Importantly, the enrichment of LIG4 to DSBs was greatly impaired by MPP8 depletion (Figure 6I,J), suggesting that MPP8 facilitates LIG4 recruitment at DSB foci. Together, these findings support a model in which MPP8 is recruited to DSBs upon DNA damage, likely by KU80 via their robust protein interactions and in turn facilitates the recruitment of ligase complex and assembly of NHEJ apparatus at DSB foci for repair.

### 2.7 MPP8 Promotes Chemoresistance of Breast Cancer In Vivo

Having demonstrated that MPP8 promotes chemoresistance of different TNBC cells by facilitating DNA DSB repair via NHEJ pathway, we set out to determine if MPP8 depletion modulates chemotherapy sensitivity of TNBC in vivo using murine tumor models. To this end, we inoculated Ctrl‐KD or MPP8‐KD MDA‐MB‐231 cells into mammary fat pads of SCID mice. After tumors reached 50 mm^3^, mice were randomized into different groups to receive Dox or PBS treatment for five weeks. In PBS‐treated groups, growth of MPP8‐KD tumors became significantly slower (*p* < 0.01) than Ctrl‐KD tumors six weeks after inoculation, consistent with our previous characterization of MPP8 as a potential oncogene. In Dox‐treated groups, MPP8‐KD tumors displayed a significantly attenuated growth (*p* = 0.001) after four weeks treatment comparing with PBS‐treated group, whereas Ctrl‐KD tumors only showed a non‐significant reduction (*p* = 0.103), suggesting that depletion of MPP8 resensitized xenografted tumors to Dox‐treatment (Figure 7A). This notion was further supported by the measurement of dissected primary tumors at the end stage (Figure 7B,C). Consistent with the pro‐EMT function of MPP8 we reported previously [8, 9], quantification of metastatic lung nodules revealed a significant reduction of metastatic capability of MPP8‐KD tumors. Intriguingly, Dox treatment further reduced lung metastases of MPP8‐KD tumors. In contrast, it increased lung metastases of Ctrl‐KD tumors (Figure 7D), a similar phenotype reported previously in MDA‐MB‐231 orthotopic model [35] and PyMT transgenic model [36]. To further assess the role of altered DNA repair capabilities in Dox sensitivity of different tumors, we examined the levels of DNA DSBs by immunohistochemistry (IHC). As shown in Figure 7E, both Dox‐treated Ctrl‐KD and MPP8‐KD tumors showed an elevated γH2AX level. However, a substantially increase of γH2AX was detected in MPP8‐KD tumors compared with Ctrl‐KD tumors. We also detected a similarly increased cleaved Caspase3 level in MPP8‐KD tumors. Western blot analysis of dissected tumors further confirmed these changes (Figure 7F). Collectively, these results are consistent with our in vitro analyses and suggest that depletion of MPP8 reduces DNA repair capability of xenografted TNBC in vivo and thereby resensitizes tumors to Dox treatment.

**FIGURE 7:**
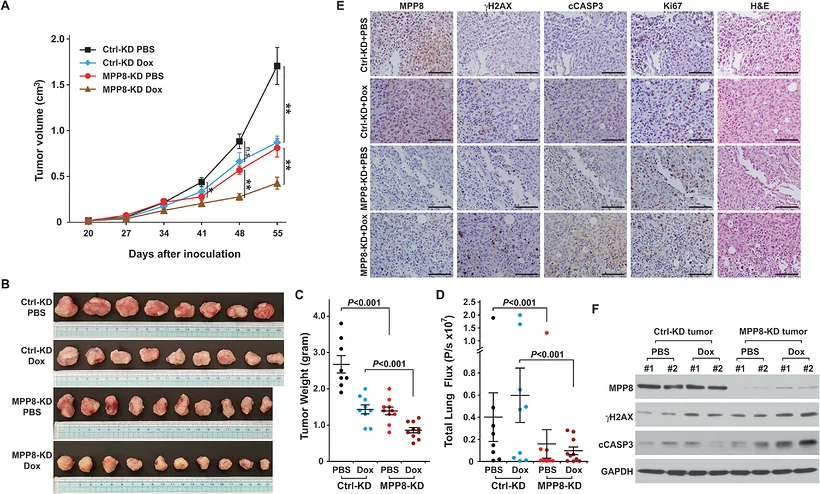
MPP8 promotes chemoresistance of TNBC to doxorubicin in xenograft models. (A) Primary tumor growth (mean±SEM) measured by a caliper in SCID mice orthotopically xenografted with control‐ or MPP8‐ KD MDA‐MB‐231 cells (*n* = 8–10) with PBS or Dox treatment. *p < 0.01, **p < 0.005. (B) Images of dissected end‐stage control and MPP8‐KD primary tumors after PBS or Dox treatment. (C) Weight quantification of dissected end‐stage control and MPP8‐KD primary tumors after PBS or Dox treatment (mean±SEM). (D) Bioluminescence quantification of dissected lungs from mice bearing control‐ and MPP8‐KD tumors after PBS or Dox treatment (mean±SEM). (E) Images of end‐stage control and MPP8‐KD primary tumor sections stained with haematoxylin and eosin (H&E) or antibody against MPP8, γH2AX, cleaved Caspase3 and Ki67. Scale bars are 100 µm. (F) Western blot analysis of two end‐stage primary tumors from control‐ and MPP8‐ KD MDA‐MB‐231 xenograft models using indicated antibodies.

## 3 Discussion

In this study, we demonstrate that MPP8 could act as an NHEJ adaptor independent of its canonical H3K9me3 binding and transcription repression function. In this scenario, MPP8 is dynamically recruited to DSBs upon DNA damage, presumably by KU80 through its robust interactions with MPP8 C‐terminal domain. The enrichment of MPP8 at DSBs further facilitates the recruitment of ligase complex via XLF, resulting in a stabilized assembly of NHEJ repair apparatus to expedite repair progression. Considering that NHEJ is the dominant DSB repair pathway in human cells, we propose that this non‐canonical role of MPP8 in DSB repair serves as another regulatory layer to maintain genome integrity, in addition to H3K9me3 binding‐related mechanisms. In cancer cells, however, such a function of MPP8 could enhance overall DSB repair capacity and render resistance to genotoxic reagents such as doxorubicin and IR.

Since we characterized MPP8 as a H3K9me3 binding protein, most MPP8 studies have been focused on transcription repression mediated by its N‐terminal chromodomain. MPP8 also forms the human silence hub (HUSH) complex with TASOR and PPHLN1, in which MPP8 binds to H3K9me3 and recruits SETDB1 to propagate H3K9me3 for epigenetic silencing of different viral elements and endogenous LINE‐1s [10, 11, 12]. Although both N‐terminal chromodomain and C‐terminus of MPP8 are required for the recruitment of HUSH complex to endogenous LINE‐1s, a recent study revealed that Mpp8 C‐terminus is necessary to maintain epigenetic silencing of LINE‐1s and self‐renewal of ground‐state pluripotent mESCs [10]. Other than that, function of the largely uncharacterized MPP8 C‐terminus remains elusive. Here, we discovered that MPP8 C‐terminus could function as a scaffold to facilitate association of DNAPK and ligase complexes at DSBs to promote DNA repair via NHEJ pathway through its interactions with KU80 and XLF. Although our results suggest that DNA and RNA are not involved in these interactions, it is unclear if they are mediated by intermediate protein(s). The computationally modeled MPP8_525‐860_‐KU80 and MPP8_525‐860_‐XLF complexes suggest that MPP8 may directly interact with KU80 and XLF. However, such a possibility needs to be evaluated by further investigations, such as mutagenesis, binding, and structural analyses. In addition, utilization of only MPP8 C‐terminus (525–860aa) and 1:1 molecular ratio of these two modeled complexes limited their application for in‐depth analyses of MPP8‐assisted assembly of NHEJ repair apparatus, which involves multiple dimerization and complex formation. Intriguingly, MPP8 also forms a multimer via the same C‐terminus (525–860aa, data not shown) in our IP‐Western analysis, which we believe could provide additional interfaces to facilitate multiple simultaneous protein‐protein interactions for the assembly of NHEJ apparatus at DSB foci.

MPP8 and HUSH complex mostly represses evolutionarily young, primate‐specific, and retrotransposition‐competent LINE‐1s through H3K9me3 [13, 14, 37]. In AML cells, MPP8 depletion increases LINE‐1 retrotransposition, leading to increased γH2AX level, p21 upregulation and growth defects [13]. Although this elegant study suggests that MPP8 loss could create DSBs via LINE‐1 reactivation and retrotransposition, our comet assays and γH2AX measurements didn't detect any meaningful differences in basal DSB/DNA damage levels between various control‐ and MPP8‐ KD cells. While Mpp8 and HUSH complex are essential for LINE‐1 silencing in hypomethylated naïve mESCs [10], DNA methylation plays the crucial role in committed somatic cells. Therefore, it is possible that epigenetic silencing of young, retro‐transposable LINE‐1s are maintained or compensated by DNA methylation in different stable MPP8‐KD tumor cells used in our assays. Considering that repetitive sequences including LINE‐1s and Alu elements often become hypomethylated in various cancer cells, we are actively investigating the possible role of MPP8‐LINE‐1 axle in genome stability in TNBC cells and chemoresistance. Meanwhile, another recent study showed that MPP8 knockdown in 293 cells led to a modest increase of γH2AX level. Intriguingly, etoposide‐induced γH2AX foci were mostly cleared in control‐KD cells after 3 h recovery but largely sustained in MPP8‐KD cells even after 6 h recovery [37]. These results are consistent with our findings derived from different tumor cells and further support that MPP8 plays a pivotal role in DSB repair progression. Given that expression of H3K9me3 binding deficient MPP8‐W80A restored chemo‐ and radio‐ resistance of various MPP8‐KD TNBC cells, we speculate that direct involvement of MPP8 in NHEJ repair pathway could play a more pronounced role in resistance to genotoxic reagents than suppressing LINE‐1 expression and retrotransposition.

H3K9me3 accumulates at DSB foci at very early stage of DDR and serves as a docking platform for various chromatin regulating proteins, including chromodomain‐containing TIP60, HP1, and SUV39H1 to transiently regulate local chromatin dynamics for repair [38, 39]. It raises an intriguing question—why is MPP8 not involved in DSB repair similarly via its chromodomain? It has been reported that Mpp8 genomic targeting wa not affected by Suv39h1/h2 double knockout in mESCs, indicating that Mpp8 is not recruited to genomic regions enriched with Suv39h1/h2‐catalyzed H3K9me3 [10]. It is also known that efficient SUV39H1 recruitment by TIP60 at DSB foci and sequential propagation of H3K9me3 via HP1‐SUV39H1 reader‐writer cycle are important steps to activate ATM for DDR [39]. Unlike HP1, we did not detect MPP8 interactions with either TIP60 [9] or SUV39H1 [8]. Therefore, one possible explanation is that the absence of necessary protein interactions and different chromatin localization exclude MPP8 from TIP60‐SUV39H‐HP1 mediated DSB repair pathway. Considering that H3K9me3 binding by MPP8 chromodomain is much stronger than HP1 (0.14 µm vs. 10 µm in Kd) [40], we speculate that such a stable binding may also impose an obstacle on rapid chromatin relaxation around DSBs after the initial compaction, which provides DNA accessibility to facilitate repair processes.

As an indispensable component for robust NHEJ, XLF forms a homodimer to not only scaffold DNAPK complex and ligase complex at DSBs but also bridge assembled protein‐DNA complexes at both ends [41, 42]. In the core NHEJ supercomplexes, XLF homodimer forms a head‐to‐head interaction with XRCC4 and interacts with KU70 and KU80 via its middle domain and C‐terminal X‐KBM respectively [41, 43]. However, XLF‐KU70/80 interactions are relatively weak and may only occur when other NHEJ factors are present [41]. Therefore, our findings suggest that the presence of MPP8 at DSBs could stabilize weak XLF‐KU70/80 interactions and facilitate the assembly of NHEJ supercomplexes. Furthermore, recent structural studies reveal that the DNA ends are synapsed in a domain swap and an XLF‐mediated long‐range complex which needs to be further progressed into a short‐range synaptic complex where DNA ends are close enough for ligation [41, 42]. Intriguingly, transition into the short‐range synaptic complex requires formation of the XLF‐mediated long‐range synaptic complex [44]. Although the possible involvement of MPP8 in maturation of synaptic complexes warrants further investigation, we speculate that MPP8‐stabilized XLF‐KU70/80 interactions may also facilitate formation of the XLF‐dependent long‐range complex and thus contribute to its progression into the short‐range synaptic complex via its interactions with XLF. In these scenarios, we hypothesize that DNA damage‐induced MPP8‐XLF interaction could play an important role. While the mechanism underlying this interaction is being investigated, it is not clear if DNA‐PKcs kinase activity plays a role, even though MPP8 does not contain any conserved S/T‐Q or S/T‐ψ‐D/E motif and XLF‐S245/S251 phosphorylation does not affect NHEJ repair [45]. Meanwhile, our data suggests that formed XLF‐MPP8 interaction facilitates XLF nuclear retention upon DNA damage. Such retention may also counteract XLF‐T181 phosphorylation to prevent XLF dissociation from ligase complex [30]. Together, we propose MPP8 as a new NHEJ accessory factor which augments the repair capacity of the core NHEJ complex by regulating XLF at multiple molecular layers. Therefore, MPP8 level may be used as an indicator to predict possible outcomes of TNBC genotoxic chemotherapy.

## 4 Experimental Section

### 4.1 Cell Lines, Antibodies, and Inhibitors

MDA‐MD‐231, Hs578T, MDA‐MB‐468, MCF7, U‐2 OS, HeLa‐S3, 293T (ATCC), as well as MDA‐MB‐231‐D3H2LN (Xenogen) and NHEJ and HR reporter 293–140c and 293–97A [23] cells were cultured in DMEM with 10% FBS and penicillin/streptomycin (Corning). MPP8 antibody was affinity‐purified from rabbit serum against purified antigens (1‐188aa) [8] while other antibodies were from commercial sources, including Cell Signaling (PARP1, cleaved Caspase3, γH2AX, KU70, KU80, Lamin B, LIG4, XLF), Abcam (Ki67, LDHB), Sigma (Actin, Flag‐M2), Santa Cruz (XLF, HA), Proteintech (GAPDH, XRCC4), Upstate (γH2AX), GeneTex (LIG4) and JacksonImmuno (IgG and secondary antibodies). Doxorubicin, Shield‐1, and 4‐OHT were from Sigma and Cayman Chemical.

### 4.2 Mouse and Xenografts

Animal care and experiments were approved by the Institutional Animal Care and Use Committee (IACUC). For xenografts, ∼2 × 10^5^ control‐KD and MPP8‐KD MDA‐MB‐231‐D3H2LN cells were resuspended in 50% Matrigel and orthotopically injected into the fourth inguinal mammary fat pad of randomized female SCID mice (*C.B‐Igh‐1bIcrTac‐Prkdcscid/Ros*, 8wks). After primary tumors reached 50 mm^3^, mice were randomized into different groups to receive Dox (1 mg/kg body weight) or PBS treatment twice a week for 5 weeks while tumor growth was monitored by caliper measurement. For in vivo bioluminescence imaging (IVIS Spectrum imager), mice were injected (i.p.) with 10 µL/g body weight D‐luciferin (Goldbio, 15 mg/mL in PBS). At the endpoint, primary tumors and lungs were harvested for ex vivo imaging. Bioluminescence was quantified (Living Image software, PerkinElmer) as total flux (photos/sec) and average radiance (total flux/cm^2^/Sr) were calculated. Primary tumors were also embedded, sectioned, and stained with Hematoxylin and Eosin. For immunohistochemistry (IHC), paraffin sections were treated with 3% H_2_O_2_ and blocked by 10% goat serum after antigen retrieval with sodium citrate. After incubating with MPP8, Ki67, H2AX or cleaved Caspase3 antibody and biotinylated second antibodies sequentially, sections were developed with diaminobenzidine (DAB) and mounted.

### 4.3 Comet Assays

Control‐ and MPP8‐ KD MDA‐MB‐231 were seeded overnight before being treated with DMSO, Doxorubicin (1 µm, 1 h) or γ‐irradiated (2 Gy). Cells were then harvested at 0, 4, and 24 h after treatment while the neutral and the alkaline comet assays were performed as previously described [46]. DNA tail moment was quantified using ImageJ.

### 4.4 Immunofluorescence and Proximity Ligation Assay

To process immunostaining, cells were seeded on glass coverslip overnight before being treated with DMSO, γ‐irradiated (2 Gy) or Doxorubicin (1 µm for 1 h). Drug was washed out while cells were allowed to recover for different times before being pre‐extracted with Triton‐X100 (20 mm HEPES pH 7.4, 50 mm NaCl, 3 mm MgCl_2_, 300 mm sucrose, 0.5% Triton‐X100) for 10 min and fixed in 4% paraformaldehyde for 15 min at RT. For immunofluorescence, cells were blocked with 5% goat serum, incubated with primary antibodies overnight at 4°C, washed three times to remove non‐specific binding, incubated with secondary antibody for 30 min at RT, and stained with 4′,6‐diamidino‐2‐phenylindole (DAPI). For PLA, fixed cells were blocked with Duolink Blocking Solution (Sigma) and incubated with primary antibody in Duolink Antibody diluent overnight at 4°C. Proximity ligation was completed according to the manufacturer's protocol (Sigma), while images were obtained and formed PLA foci was counted. More than 300 cells were analyzed in each group.

### 4.5 I‐PpoI‐Mediated DSB Repair and Chromatin IP (ChIP) Assays

U‐2 OS cells stably expressing dd‐ER‐I‐PpoI were generated by infection of retrovirus derived from pBABE‐ddIPpoI (Addgene #49052) [34], puromycin selection, and characterization of single isolated clones. To induce DSBs, Shield‐1 was added into culture media for 3 h followed by 4‐OHT for additional 15 min incubation before both drugs being washed out. To quantify DSB repair, cells were collected at different timepoints to extract genomic DNA while qPCR was carried out using primers spanning defined I‐PpoI sites as prior described [47, 48]. ChIP assays were carried out as we described previously [49] with some modifications. Briefly, chromatin was cross‐linked with 1% formaldehyde at different timepoints after DSB induction sheared to an average size of 800 bp using a Bioruptor. ChIP assays were carried out using normal rabbit IgG, anti‐MPP8 (ref [8]) or anti‐LIG4 antibody (GeneTex GTX55592) and magnetic beads (Pierce) followed by extensive washing. Reverse cross‐linked ChIPed DNA was quantified by qPCR using primers described previously [47, 48].

### 4.6 Statistical Analysis

For caspase activity assay, clonogenic assay, γH2Ax foci quantification, NHEJ and HR reporter assay and plasmid integration assay, the *p*‐value was determined using two‐tailed Student's *t*‐test or Welch's *t*‐test with the standard deviation (±SD). For PLA, the comet assay and quantification of mouse tumors (volume, weight, and total lung flux), the *p*‐value was calculated using Student's *t*‐test or Welch's *t*‐test with the standard error of the mean (±SEM). A *p*‐value less than 0.05 is considered statistically significant. All statistical analyses were carried out using GraphPad Prism version 10.

### 4.7 Other Assays

Details for other assays including knockdown, Caspase‐3 activity, clonogenic assay, MPP8 protein complex purification, immunoprecipitation, chromatin fractionation, NHEJ and HR reporter assays, plasmid integration assay, subcellular fractionation, etc., are available in the supporting information.

## Author Contributions

J.F. conceived the project, designed the experiments, and wrote the manuscript. R.K. carried out the experiments and analyzed data.

## Conflicts of Interest

The authors declare no conflicts of interest.

## Supporting information




**Supporting File**: advs78157‐sup‐0001‐SuppMat.pdf.

## Data Availability

The data that support the findings of this study are available on request from the corresponding author. The data are not publicly available due to privacy or ethical restrictions.
