# Supplementary material for: MPP8 Facilitates DNA DSB Repair via NHEJ to Promote Chemoresistance of TNBC Independent of Methyl‐H3K9 Binding
Source: Adv Sci (Weinh). 2026 Oct 6:e78157. Online ahead of print. doi: 10.1002/advs.78157 (PMC13643101; doi:10.1002/advs.78157)
Supplement: Supplementary file 1 [file ADVS-9999-e78157-s001.pdf]

Supplemental Data

MPP8 facilitates DNA DSB repair via NHEJ to promote chemoresistance of TNBC independent of methyl-H3K9 binding

Rupa Kumari[1] and Jia Fang[1*]

[1]Department of Pharmacology & Therapeutics, Roswell Park Comprehensive Cancer Center, Elm & Carlton Streets, Buffalo, NY 14263

[*]Correspondence and requests for materials should be addressed:

J.F. (Jia.Fang@RoswellPark.org)

Phone: 716-845-2926

Fax: 716-845-8857

Competing Interests statement: The authors declare no potential competing interests.

Kumari and Fang, Supplemental Figure S1

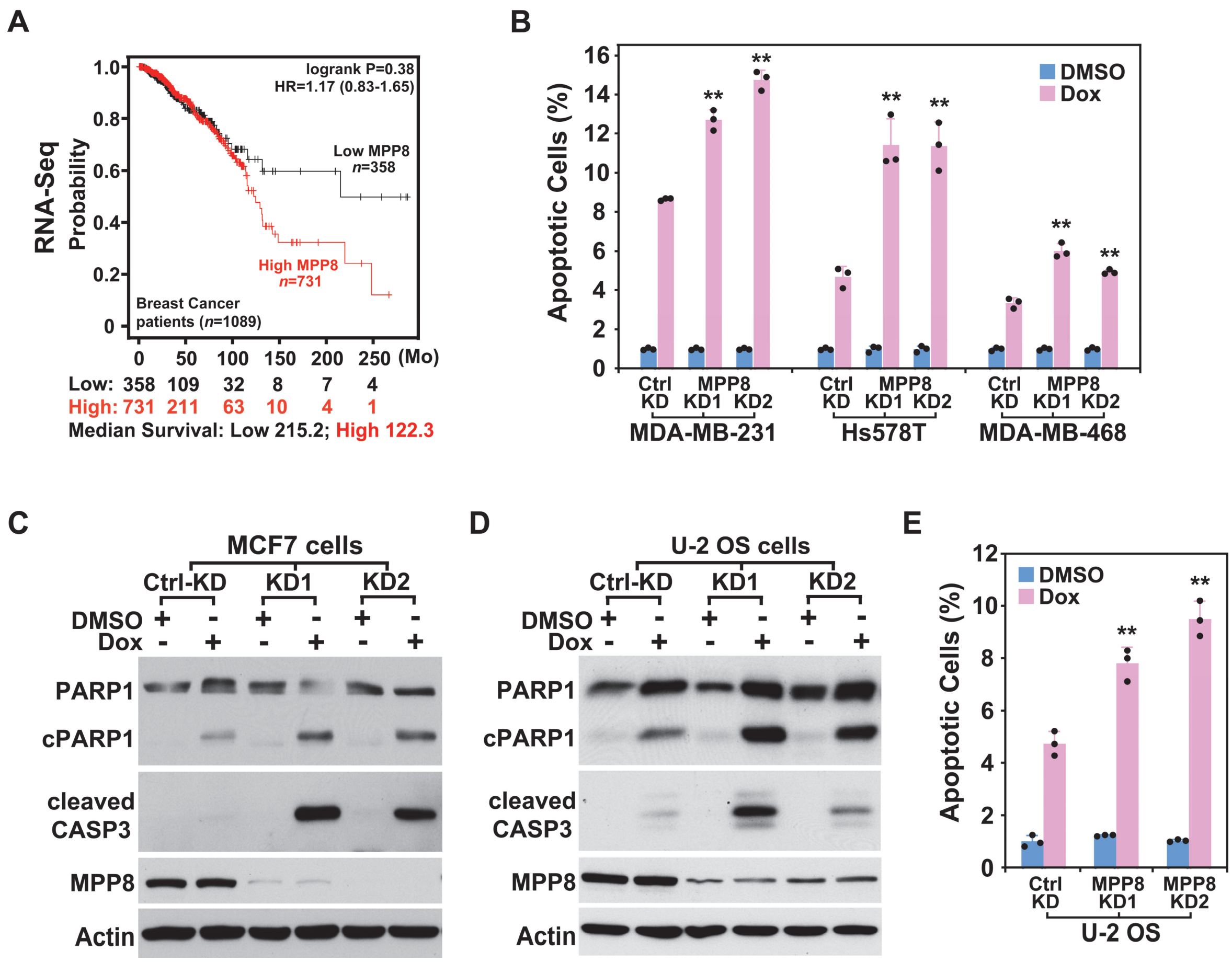

Supplemental Figure S1. MPP8 promotes chemoresistance of tumor cells to Dox. (A) Kaplan-Meier plots for overall survival of breast cancer patients stratified by MPP8 expression level with *P* values from univariate (log-rank). **(B)** Apoptotic population of control- and two MPP8- KD MDA-MB-231, Hs578T and MDA-MB-468 cells after DMSO or Dox treatment. Results are from 3 independent experiments (mean±s.d.) while DMSO treated cells were normalized as 1. ** indicates *P*<0.01. **(C-D)** Western blot analyses of whole cell extracts (WCE) derived from different control- and MPP8- KD MCF7 **(C)** and U-2 OS **(D)** cells after DMSO or Dox treatment using indicated antibodies. **(E)** Apoptotic population of control and two MPP8- KD U-2 OS cells after DMSO or Dox treatment. Results are from 3 independent experiments (mean±s.d.) while DMSO treated cells were normalized as 1. ** indicates *P*<0.01.

Kumari and Fang, Supplemental Figure S2

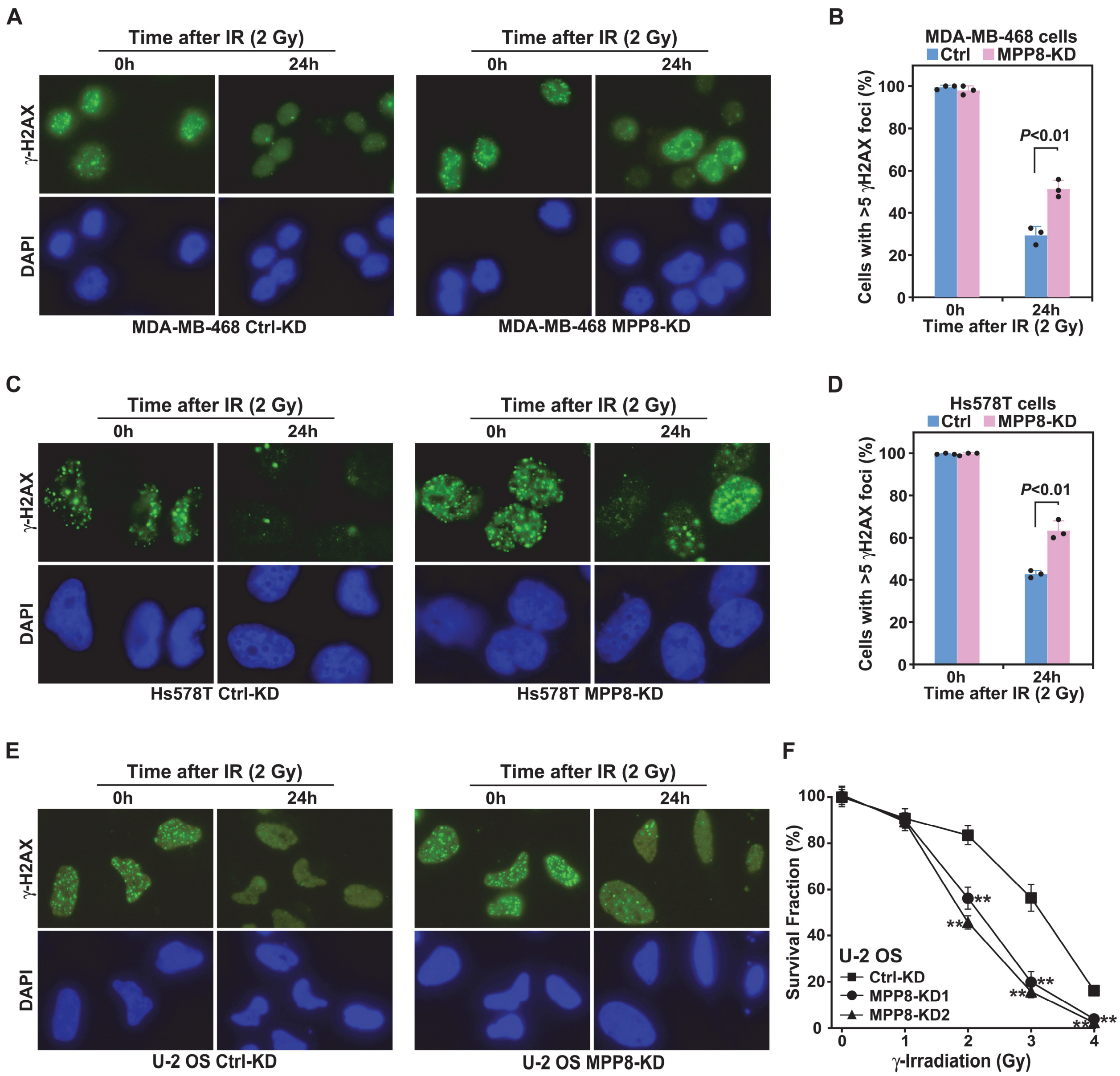

Supplemental Figure S2. MPP8 promotes repair of DNA DSBs. (A) Representative images of control-KD and MPP8-KD MDA-MB-468 cells stained with γH2AX (green) and DAPI (blue) 0h and 24h after IR treatment. **(B)** Quantification of control-KD and MPP8-KD MDA-MB-468 cells with γH2AX foci (>5/cell) after IR treatment. Results are mean±s.d. from 3 independent experiments and 100-150 cells were analyzed for each group. **(C)** Representative images of γH2AX- (green) and DAPI- (blue) stained control-KD and MPP8-KD Hs578T cells 0h and 24h

after IR treatment. **(D)** Quantification of control-KD and MPP8-KD Hs578T cells with γH2AX foci (>5/cell) after IR treatment. Results are mean±s.d. from 3 independent experiments and 100-150 cells were analyzed for each group. **(E)** Representative images of γH2AX (green) and DAPI (blue) stained control-KD and MPP8-KD U-2 OS cells 0h and 24h after IR treatment. **(F)** Clonogenicity assay of control- and two MPP8- KD U-2 OS cells with increasing doses of IR. Colonies were quantified after 14 days and colony numbers without IR were normalized as 100%. Results are mean±s.d. from 3 independent experiments. ** indicates $P<0.01$.

Kumari and Fang, Supplemental Figure S3

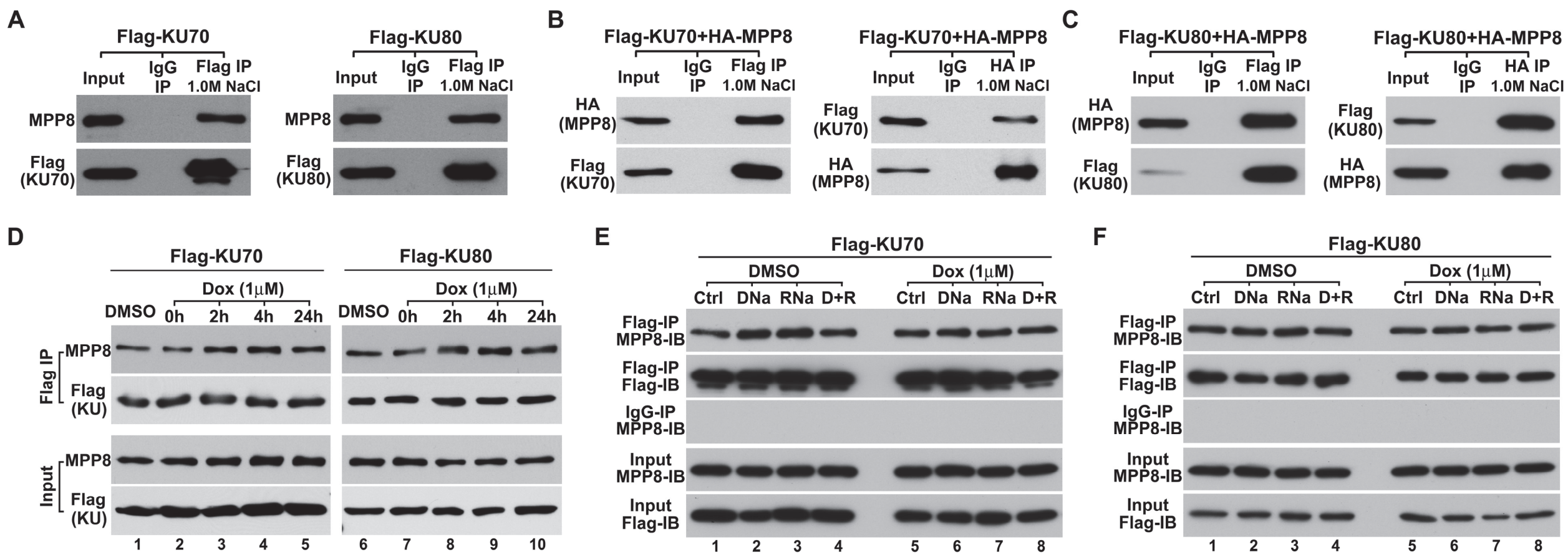

Supplemental Figure S3. MPP8 interacts with KU70 and KU80. (A) IP-western analysis of protein interactions of endogenous MPP8 with exogenously expressed Flag-KU70 (left) or Flag-KU80 (right) and in 293T cells using indicated antibodies. **(B)** IP-western analysis of protein interactions between exogenously expressed Flag-KU70 and HA-MPP8 in MPP8-KD 293T cells. IP was performed with Flag (left) or HA (right) antibody while western blots were probed with indicated antibodies. **(C)** IP-western analysis of protein interactions between exogenously expressed Flag-KU80 and HA-MPP8 in MPP8-KD 293T cells. IP was performed with Flag (left) or HA (right) antibody, and western blots were probed with indicated antibodies. **(D)** IP-western analysis of protein interactions of endogenous MPP8 with exogenously expressed Flag-KU70 (left) or Flag-KU80 (right) after DMSO or Dox treatment at indicated time points. Protein levels in input samples were also examined by western blot. **(E-F)** IP-western analysis of protein interactions of endogenous MPP8 with exogenously expressed Flag-KU70 **(E)** or Flag-KU80 **(F)** 4h after DMSO or Dox treatment. Cell lysates derived from different transfected cells were pre-treated with 100U/mL DNase I (DNa), 50μg/mL RNase A (RNa) or both (D+R) before IP. Protein levels in input samples were also examined by western blot.

Kumari and Fang, Supplemental Figure S4

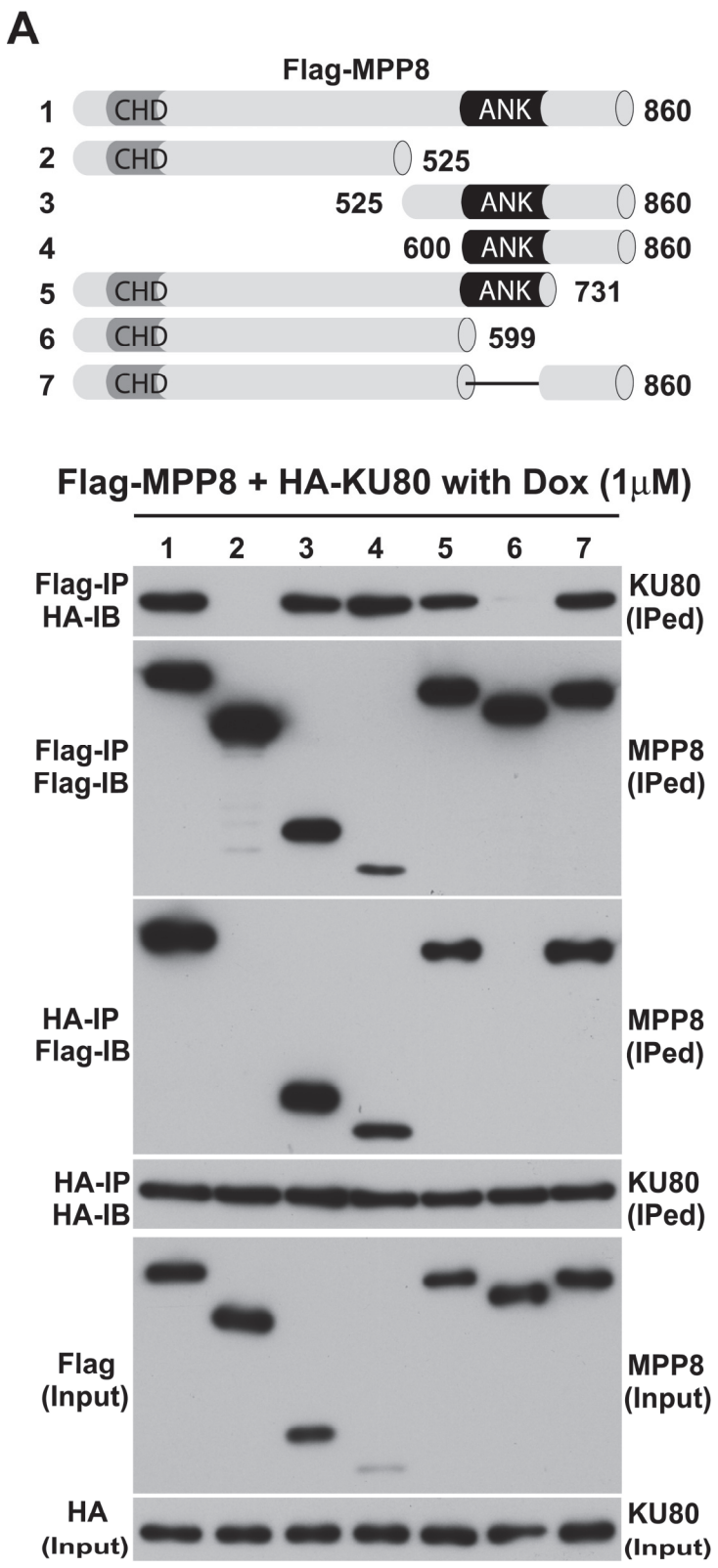

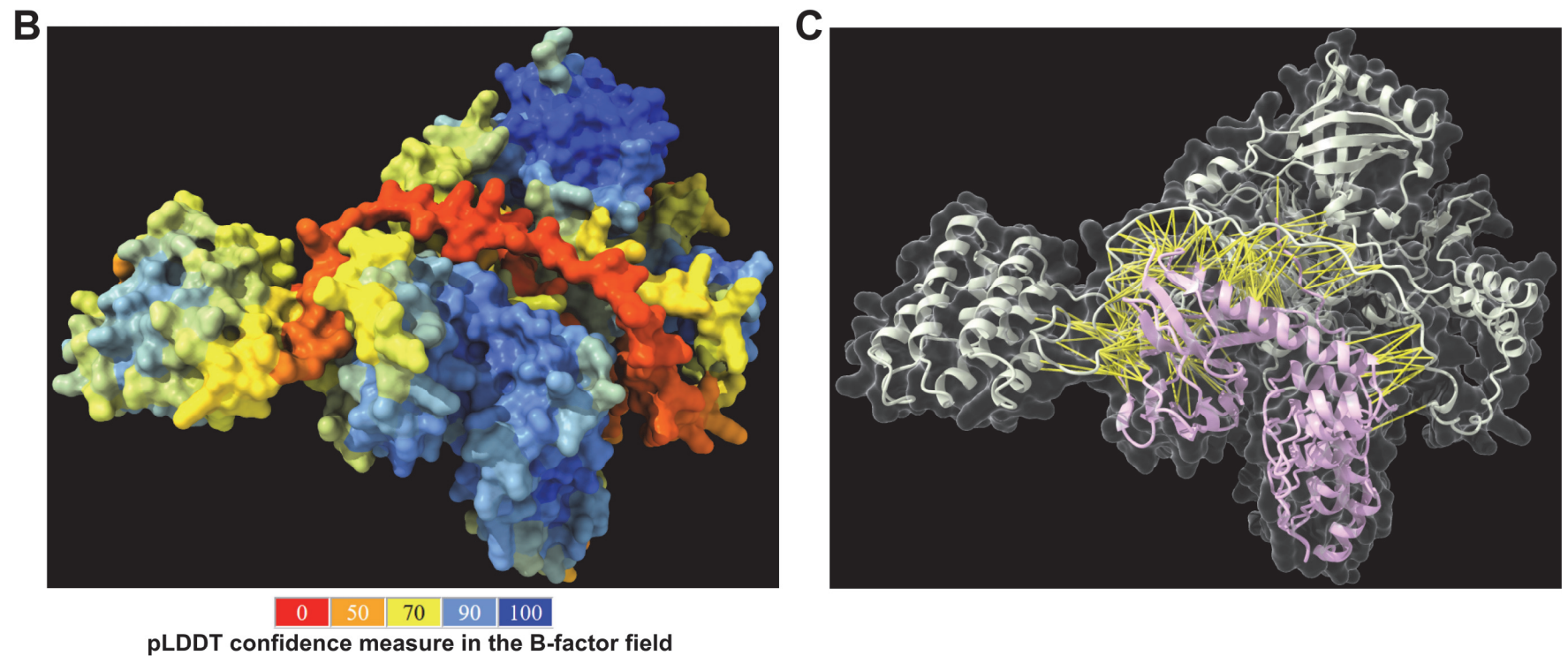

D Ignore distances between atoms separated by 4 bonds or less
Detect intra-residue distances: False; Detect intra-molecule distances: True

31 distances

MPP8	Atom1	KU80	Atom2	Distance
ARG 741	NH2	GLY 573	O	2.761
ARG 741	NH1	PHE 576	CB	2.784
LEU 781	CD2	LEU 581	CD2	2.974
ILE 795	CD1	ARG 315	NH2	0.987
ILE 795	CD1	ARG 315	CZ	2.032
ILE 795	CG1	ARG 315	NH2	2.473
ILE 795	CD1	ARG 315	NH1	2.799
ILE 795	CD1	ARG 315	NE	2.98
PRO 801	CG	ASP 299	CB	1.098
PRO 801	CG	ASP 299	CG	1.787
PRO 801	CD	ASP 299	CB	1.983
PRO 801	CG	ASP 299	OD2	2.111
PRO 801	CB	ASP 299	CB	2.403
PRO 801	CG	ASP 299	CA	2.498
PRO 801	CD	ASP 299	OD2	2.586
PRO 801	CD	ASP 299	CG	2.683
PRO 801	CG	ASP 299	OD1	2.901

MPP8	Atom1	KU80	Atom2	Distance
VAL 817	CG2	LEU 581	CD2	1.576
VAL 817	CG1	LEU 581	CG	1.703
VAL 817	CG1	LEU 581	CD2	2.022
VAL 817	CB	LEU 581	CD2	2.045
VAL 817	CG1	LEU 581	CD1	2.629
VAL 817	CB	LEU 581	CG	2.65
VAL 817	CG2	LEU 581	CG	2.665
VAL 817	CG1	LEU 581	CB	2.97
SER 826	CB	LEU 581	O	1.912
SER 826	OG	LEU 581	O	2.707
SER 826	CB	LEU 581	C	2.784
SER 826	OG	ALA 582	CA	2.394
SER 826	OG	ALA 582	N	2.475
SER 826	CB	ALA 582	N	2.871

Supplemental Figure S4. MPP8 interacts with KU80 through its C-terminal domain. (A) Different Flag-MPP8 deletions (top) were co-expressed with HA-KU80 in MPP8-KD 293T cells followed by Dox treatment (1µM 1h). Cells were then lysed 4h after Dox treatment for Flag or HA IP and Western blot analysis (bottom). Protein levels in input samples were also examined by Western blot. **(B)** Alphafold3 predicted structure of $MPP8_{525\text{-}860}$-KU80 protein complex colored by pLDDT confidence measured in the B-factor field. **(C)** Modeled structure of $MPP8_{525\text{-}860}$-KU80 complex with predicted intermolecular contacts (yellow) between $MPP8_{525\text{-}860}$ (light pink) and KU80 (light khaki) at distance 8. **(D)** Identified amino acids and their atom groups being involved in predicted intermolecular interactions at distance 3 in modeled $MPP8_{525\text{-}860}$-KU80 complex.

Kumari and Fang, Supplemental Figure S5

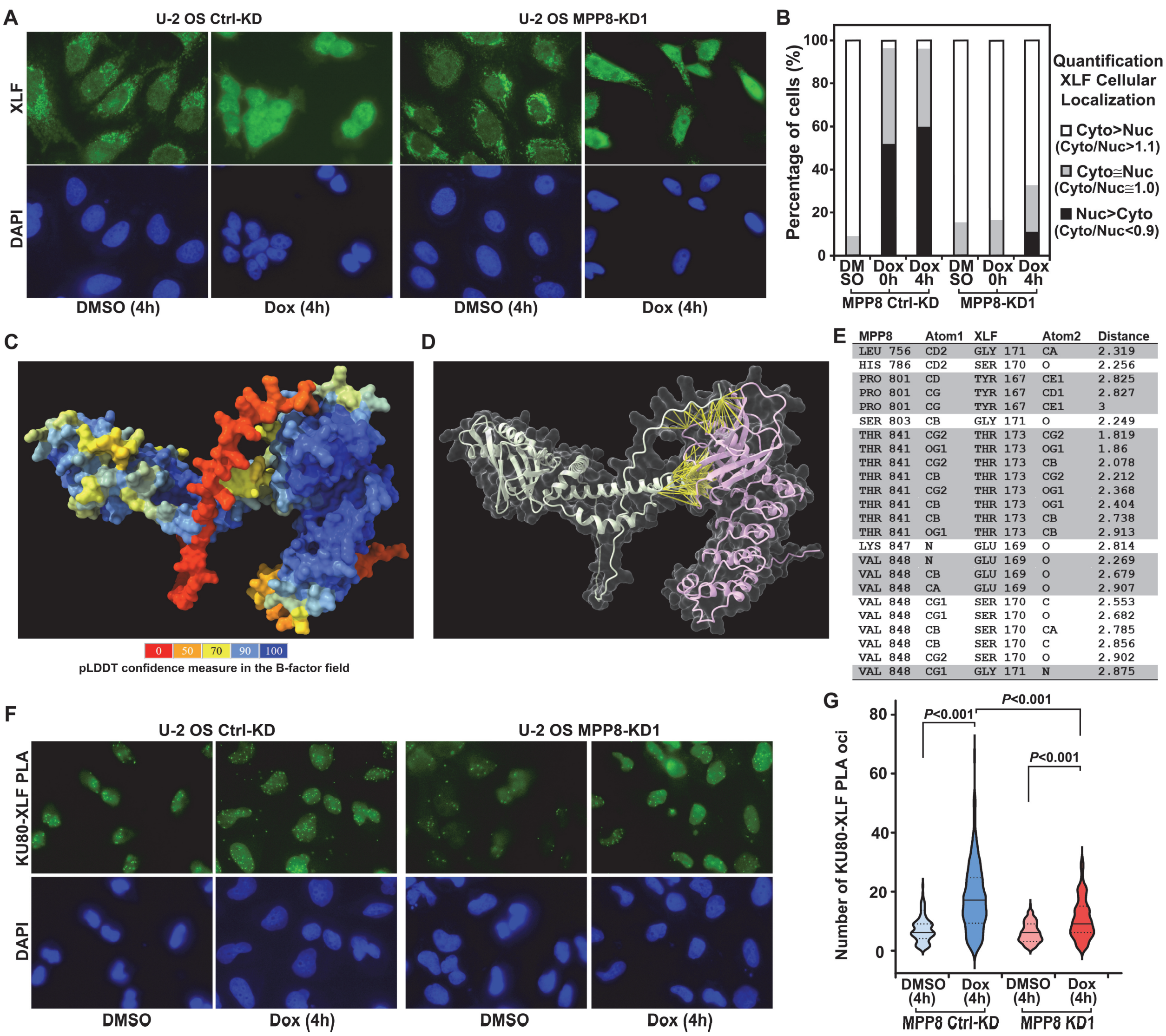

MPP8	Atom1	XLF	Atom2	Distance
LEU 756	CD2	GLY 171	CA	2.319
HIS 786	CD2	SER 170	O	2.256
PRO 801	CD	TYR 167	CE1	2.825
PRO 801	CG	TYR 167	CD1	2.827
PRO 801	CG	TYR 167	CE1	3
SER 803	CB	GLY 171	O	2.249
THR 841	CG2	THR 173	CG2	1.819
THR 841	OG1	THR 173	OG1	1.86
THR 841	CG2	THR 173	CB	2.078
THR 841	CB	THR 173	CG2	2.212
THR 841	CG2	THR 173	OG1	2.368
THR 841	CB	THR 173	OG1	2.404
THR 841	CB	THR 173	CB	2.738
THR 841	OG1	THR 173	CB	2.913
LYS 847	N	GLU 169	O	2.814
VAL 848	N	GLU 169	O	2.269
VAL 848	CB	GLU 169	O	2.679
VAL 848	CA	GLU 169	O	2.907
VAL 848	CG1	SER 170	C	2.553
VAL 848	CG1	SER 170	O	2.682
VAL 848	CB	SER 170	CA	2.785
VAL 848	CB	SER 170	C	2.856
VAL 848	CG2	SER 170	O	2.902
VAL 848	CG1	GLY 171	N	2.875

Supplemental Figure S5. MPP8 facilitates assembly of NHEJ repair apparatus via XLF after Dox treatment. (A) Immunostaining of XLF (green) and DAPI (blue) in control-KD (left) and MPP8-KD (right) U-2 OS cells 4h after DMSO or Dox treatment. **(B)** Quantification of XLF subcellular localization in control- and MPP8- KD U-2 OS cells after DMSO or Dox treatment. The staining density of cytoplasmic and nuclear XLF in each cell was quantified while the ratios of cytoplasmic/nuclear XLF were indicated in the bar graph. 200~300 cells in each group were analyzed. **(C)** Alphafold3 predicted structure of $MPP8_{525\text{-}860}$-XLF protein complex colored by

pLDDT confidence measured in the B-factor field. **(D)** Structure of modeled $MPP8_{525\text{-}860}$-XLF complex with predicted intermolecular contacts (yellow) between $MPP8_{525\text{-}860}$ (light pink) and XLF (light khaki) at distance 8. **(E)** Identified amino acids and their atom groups that mediate predicted intermolecular interaction at distance 3 in the modeled $MPP8_{525\text{-}860}$-XLF complex. **(F)** In situ KU80-XLF interactions in control-KD and MPP8-KD U-2 OS cells were measured by PLA foci 4h after DMSO or Dox treatment (green). The nucleus was co-stained by DAPI (blue). **(G)** Quantification of KU80-XLF PLA foci. Results are mean±SEM and more than 300 cells were analyzed in each group.

Kumari and Fang, Supplemental Figure S6

A

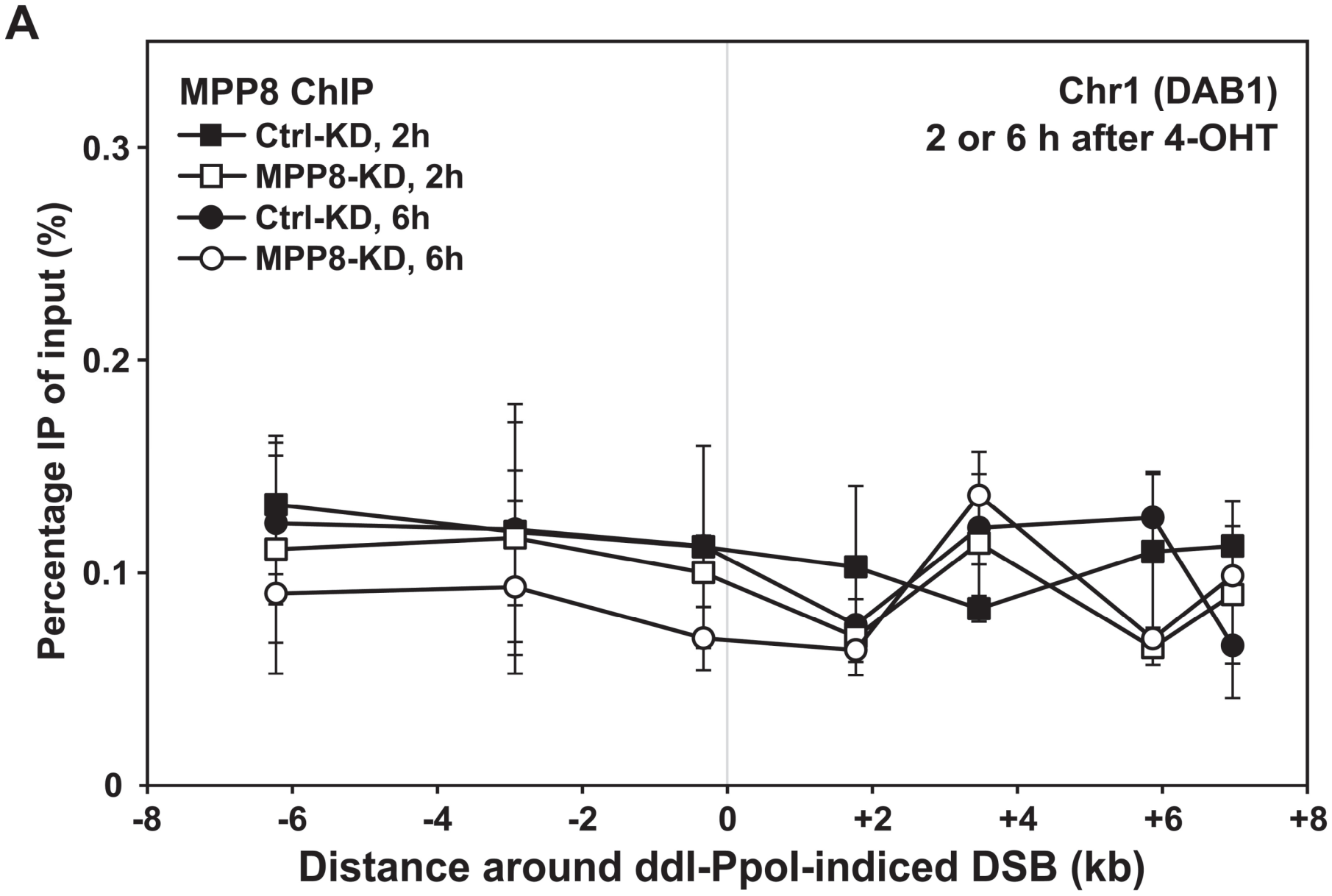

Supplemental Figure S6. Control ChIP-qPCR analysis around the I-PpoI site within DAB1 gene in control- and MPP8- KD cells. Quantified proportion of input DNA and IgG enrichment from ChIP-qPCR analysis at 2h and 6h after 4-OHT addition. Results represent mean±s.d. from 3 independent experiments.

Supplemental Experimental Procedures

Stable MPP8 knockdown

To target human MPP8, five shRNAs were designed and tested individually for knockdown efficiency determined by western blot. Two shRNAs with the best knockdown efficiency were cloned into pLenti-puro, pLenti-hygro or pLenti-blast vector. Lentivirus generation, infection and selection were described previously[1,2].

shRNA1 targeting MPP8: 5'-GCAGAGGAATCAAGACAGA-3'

shRNA2 targeting MPP8: 5'-GTGCTGCAGAGTTAGAGAA-3'

Control shRNA: 5'-ACTAAGCGAGAATTGAGGC-3'

Caspase-3 (DEVDase) activity assay

Different tumor cells were lysed with NP40 lysis buffer (50mM HEPES pH7.4, 150mM NaCl, 1mM EDTA, 1.0% NP40) and incubated with 125uM DEVD-AFC (caspase-3 substrate) at 37°C for 60 min in caspase activity assay buffer (50mM HEPES pH7.4, 100mM NaCl, 0.1% CHAPS, 1mM EDTA, 10mM DTT, 10% glycerol). Fluorescence intensity was detected using a Synergy microplate reader at excitation and emission wavelengths of 400nm and 508nm, respectively. Arbitrary fluorescence units were normalized with protein content of cell lysates and presented as fold change compared to control groups.

Clonogenicity assays:

Different tumor cells were seeded overnight before being treated with increased dose of doxorubicin for 1h or γ-irradiation. Cells were then harvested and reseeded into a 6-well plate at 1000 cells per well density.

Media was changed at day 7 and day 12 while formed colonies were fixed using 10% NBF and stained with 0.5% crystal violet at day 15 for quantification.

Immunoprecipitation

HEK-293T cells were first transfected with different expressing vectors using polyethylenimine (Polysciences). 48h after transfection, cells were collected and lysed with IPH buffer (50mM Tris-HCl pH8.0, 150mM NaCl, 0.5% NP40) with sonication. Cell lysates were then treated with 0.2U/μL MNase (Worthington Biochem) with added $CaCl_2$ to a final concentration of 1mM for 30 min at room temperature. Immunoprecipitations were carried out using 2μg of normal mouse IgG with 10μL of protein A agarose (Repligen) or 10μL of anti-Flag M2 agarose (Sigma). After 5 min washing with IPH buffer containing 1.0M NaCl three times and IP buffer containing 150mM NaCl one time, IPed proteins were analyzed by western blot. For DMSO and Dox treatment-based immunoprecipitations, cells were treated with DMSO or 1μM Dox for 1h in culture media about 48h after transfection. Cells were then collected at 4h or different time points after DMSO or Dox treatment and processed for IP-western analysis under the same conditions. For DNase I and RNase A treatment, cell lysates were pre-treated with 100U/mL DNaseI with added $MgCl_2$ to a final concentration of 2.5mM, 50μg/mL RNaseA or both 30 min at room temperature. EDTA was then added to a final concentration of 5mM to quench DNase I activity before cell lysates were further processed to IP-western analysis under the same condition.

Chromatin fractionation

Chromatin-associated fractions were prepared as previously described[3]. Briefly, MDA-MB-231 cells were resuspended in Buffer A (10mM HEPES pH7.9, 10mM KCl, 1.5mM MgCl2, 0.34M

Sucrose, 10% Glycerol, 1x protease inhibitor cocktails) before adding Triton X-100 to a final concentration of 0.1%. Cell lysates were then incubated on ice for 5 min followed by centrifugation at 300*g* for 2 min to collect the nuclei fractions (pellet). After washing once with Buffer A, the nuclei fractions were lysed in Buffer B (3mM EDTA, 0.2mM EGTA, 1mM DTT, 1x protease inhibitor cocktails) on ice for 5 min. The nuclei lysates were then centrifuged at 1700*g* for 4 min and the insoluble chromatin-associated fractions were collected and washed once with Buffer B before being resuspended in Laemmli buffer and sonicated (Amp 15%, time 10sec twice).

DSB repair reporter assay

HEK-293 cell lines 293-140c and 293-97A harboring an integrated EJ5 (NHEJ) and DR-GFP (HR) cassette, respectively were kindly provided by Dr. Jeremy Stark and the reporter assays were carried out as described previously[4]. Briefly, cells were co-transfected with pCBASce-I (Addgene #26477) and pmCherry-N1 vectors while flow cytometry analysis was carried out 48h or 72h after transfection to quantify GFP and RFP positive cells.

Plasmid Integration assay

Control- and MPP8- KD U-2 OS cells were seeded overnight before being transfected with 2µg gel-purified ScaI-linearized pEGFP-puro plasmid (Addgene #45561). Cells were harvested and counted 72h after transfection and then seeded into plates with culture medium with or without puromycin (2µg/mL) to form colonies. Media was replaced at day 7 and day 12 while formed colonies were fixed with 10% NBF and stained with crystal violet (0.5% in methanol) at day 15 for quantification.

Subcellular fractionation

Subcellular fractionation was carried out using conventional hypotonic approach as previously described with modifications[5]. Briefly, cells were harvested, washed with ice-cold PBS and resuspended in the cytosolic buffer (10mM HEPES pH7.4, 10mM KCl, 0.1mM EDTA, 0.1mM EGTA, 1mM DTT, 1x protease inhibitor cocktail) to swell on ice for 15 min. NP40 was then added to a final concentration of 0.6% followed by vigorous vortex for 45 sec. After a brief centrifugation (14000 rpm, 2 min), the supernatant was collected as the cytosolic fraction while the pellet (nuclei) was washed 3 times with the cytosolic buffer and lysed in the nuclear lysis buffer (10mM HEPES pH7.4, 500mM NaCl, 1mM EDTA, 1mM EGTA, 1% NP40, 1mM DTT, 1x protease inhibitor cocktail) to release the nuclear extract.

Alphafold3 modeling

Atomic models of MPP8 C-terminal domain in complexes with KU80 and XLF were predicted with alphafold3[6] using amino acid sequences of human MPP8 (525-860aa), full length KU80 and full length XLF as input. The predicted structures with color coding, intermolecular contacts and residues involved in the contact interfaces were visualized using ChimeraX[7].

Reference

1. K. Kokura, L. Sun, M. T. Bedford, and J. Fang. "Methyl-H3K9-binding protein MPP8 mediates E-cadherin gene silencing and promotes tumour cell motility and invasion." *EMBO Journal* **29** (2010): 3673-3687, https://doi.org/10.1038/emboj.2010.239
2. L. Sun, K. Kokura, V. Izumi, et al. "MPP8 and SIRT1 crosstalk in E-cadherin gene silencing and epithelial-mesenchymal transition." *EMBO Reports* **16** (2015): 689-699, https://doi.org/10.15252/embr.201439792
3. Y. Zhang, Q. He, Z. Hu, et al. "Long noncoding RNA LINP1 regulates repair of DNA double-strand breaks in triple-negative breast cancer." *Nature Structural & Molecular Biology* **23** (2016): 522-530, https://doi.org/10.1038/nsmb.3211
4. A. Gunn, and J. M. Stark. "I-SceI-based assays to examine distinct repair outcomes of mammalian chromosomal double strand breaks." *Methods in Molecular Biology* **920** (2012): 379-391, https://doi.org/10.1007/978-1-61779-998-3_27
5. E. Schreiber, P. Matthias, M. M. Muller, and W. Schaffner. "Rapid detection of octamer binding proteins with 'mini-extracts', prepared from a small number of cells." *Nucleic Acids Research* **17** (1989): 6419, https://doi.org/10.1093/nar/17.15.6419
6. J. Abramson, J. Adler, J. Dunger, et al. "Accurate structure prediction of biomolecular interactions with AlphaFold 3." *Nature* **630** (2024): 493-500, https://doi.org/10.1038/s41586-024-07487-w
7. E. C. Meng, T. D. Goddard, E. F. Pettersen, et al. "UCSF ChimeraX: Tools for structure building and analysis." *Protein Science* **32** (2023): e4792, https://doi.org/10.1002/pro.4792
